# Development and external validation of predictive models for prevalent and recurrent atrial fibrillation: a protocol for the analysis of the CATCH ME combined dataset

**DOI:** 10.1186/s12872-019-1105-4

**Published:** 2019-05-21

**Authors:** Winnie Chua, Christina L. Easter, Eduard Guasch, Alice Sitch, Barbara Casadei, Harry J. G. M. Crijns, Doreen Haase, Stéphane Hatem, Stefan Kääb, Lluis Mont, Ulrich Schotten, Moritz F. Sinner, Karla Hemming, Jonathan J. Deeks, Paulus Kirchhof, Larissa Fabritz

**Affiliations:** 1 Institute of Cardiovascular Sciences, University of Birmingham, Edgbaston, Birmingham, B15 2TT UK; 2 Institute of Applied Health Research, University of Birmingham, Birmingham, UK; 3 Hospital Clinic, IDIBAPS, University of Barcelona, Barcelona, Catalonia Spain; 4 CIBERCV, Madrid, Spain; 5 NIHR Birmingham Biomedical Research Centre, University Hospitals Birmingham NHS Foundation Trust and University of Birmingham, Birmingham, UK; 6 Radcliffe Department of Medicine, University of Oxford, Oxford, UK; 7 Cardiovascular Research Institute Maastricht (CARIM), Maastricht University, Maastricht, Netherlands; 8 Atrial Fibrillation NETwork (AFNET), Muenster, Germany; 9 IHU-ICAN Institute of Cardiometabolism and Nutrition, Paris, France; 10 Department of Medicine I, University Hospital Munich, Ludwig-Maximilians-University, Munich, Germany; 11 German Centre for Cardiovascular Research (DZHK); partner site: Munich Heart Alliance, Munich, Germany; 12 Sandwell and West Birmingham Hospitals NHS Trust, Birmingham, UK; 13 University Hospitals Birmingham NHS Foundation Trust, Birmingham, UK

**Keywords:** Atrial fibrillation, Predictive model, Combined database, Stratified therapy

## Abstract

**Background:**

Atrial fibrillation (AF) is caused by different mechanisms but current treatment strategies do not target these mechanisms. Stratified therapy based on mechanistic drivers and biomarkers of AF have the potential to improve AF prevention and management outcomes. We will integrate mechanistic insights with known pathophysiological drivers of AF in models predicting recurrent AF and prevalent AF to test hypotheses related to AF mechanisms and response to rhythm control therapy.

**Methods:**

We will harmonise and combine baseline and outcome data from 12 studies collected by six centres from the United Kingdom, Germany, France, Spain, and the Netherlands which assess prevalent AF or recurrent AF. A Delphi process and statistical selection will be used to identify candidate clinical predictors. Prediction models will be developed in patients with AF for AF recurrence and AF-related outcomes, and in patients with or without AF at baseline for prevalent AF. Models will be used to test mechanistic hypotheses and investigate the predictive value of plasma biomarkers.

**Discussion:**

This retrospective, harmonised, individual patient data analysis will use information from 12 datasets collected in five European countries. It is envisioned that the outcome of this analysis would provide a greater understanding of the factors associated with recurrent and prevalent AF, potentially allowing development of stratified approaches to prevention and therapy management.

**Electronic supplementary material:**

The online version of this article (10.1186/s12872-019-1105-4) contains supplementary material, which is available to authorized users.

## Background

Atrial fibrillation (AF) is a common multifactorial disease that often remains undiagnosed until its first complication [1, 2]. It affects 2–3% of the population with growing incidence especially in the ageing Western population [3–5], with current estimates suggesting 150,000–200,000 newly diagnosed AF patients per year worldwide [2, 6, 7]. Patients with AF are at increased risk of stroke, cardiovascular death, heart failure, and cardiovascular hospitalisations [8–12]. Although anticoagulation can now prevent most strokes in patients with AF, other cardiovascular complications remain common even in optimally-treated patients [13]. Complications are also caused by side effects of therapy, and in addition, approximately 2% of AF patients still suffer from a major bleed even on modern anticoagulants [14, 15].

Early management of AF to restore and maintain sinus rhythm for preventing cardiovascular complications is vital [16]. Unfortunately, there is little evidence guiding rhythm control therapy approaches to prevent recurrent AF. Current treatment to prevent AF from recurring is often unsuccessful, with 30–70% of patients experiencing a recurrence within a year after initiation of antiarrhythmic drug therapy or catheter ablation [17–19]. Clinical interventions aiming to prevent AF recurrence, or to maintain sinus rhythm in patients with established AF, are largely based on “trial and error” approaches [20]. As a result, effective rhythm control is difficult to achieve and complications related to recurrent AF are common. It is clear that stratified approaches to AF prevention and management are needed to improve outcomes in AF patients. As AF is caused by many different mechanisms in different patients, there is potential for markers indicating these underlying mechanistic drivers of AF to aid the selection of an optimal, individualised therapeutic approach [21, 22].

In a variety of cardiovascular conditions, blood biomarkers have been successfully used to identify patients at risk of adverse outcomes. In AF, biomarkers can identify patients at risk of developing AF (e.g. natriuretic peptides [23–25]), and patients at risk of AF-related complications (e.g. troponin and GDF-15 relating to stroke and bleeding in anticoagulated patients with AF [26–29]).

The CATCH ME consortium (www.catch-me.info) was established with the aim of identifying and validating markers reflecting the major drivers of AF in patients to provide evidence for a mechanism-based therapeutic approach. This manuscript describes the methods that will be used to integrate markers related to potential causal mechanisms into prediction models for prevalent AF and recurrent AF in a large dataset harmonised from 12 distinct studies. Our analysis will include blood biomarkers quantified from plasma of a subset of patients.

### Analytical approach

The analytical approach has three stages. In stage 1, models will be developed to predict (a) recurrent AF, (b) prevalent AF, (c) stroke, (d) cardiovascular death and (e) worsening of heart failure. These models will include clinical characteristics as predictors. In stage 2, further predictors reflecting the major drivers of AF as established in the literature as well as those identified in a differential gene expression analysis conducted by the CATCH ME consortium will be added to the model to evaluate their predictive value over and above clinical characteristics. Seven a priori hypotheses will be tested in stage 2 (see below). In stage 3, the predictive value of the blood biomarkers quantified from plasma samples in a subset of patients will be assessed.

Analyses will be undertaken in a combined dataset created by harmonising and merging individual patient-level clinical data [30] collected in 12 clinical research projects. The included studies are led by the CATCH ME consortium partners and reflect patients treated with varied treatment strategies in different health care systems. External validation of predictive models will be undertaken in a separate dataset. We will also compare the performance of the model for recurrent AF with 4 existing scores: the CHADS_2_ score [31], the HATCH score [32], the APPLE score [33], and the ATLAS score [34].

### Specific hypotheses to be tested

Different mechanisms of AF should translate into different patterns of recurrence, and therefore into different responses to rhythm control therapy [22]. The following seven a priori hypotheses were formulated based on published research.

1. The recurrence of AF differs in patients with and without a genetic or genomic predisposition to AF [35] (defined as AF occurring first in those aged < 60 or with a family history of AF). In addition, we hypothesise that sodium channel blockers are more effective in preventing AF recurrence than other antiarrhythmic drugs in patients with a genetic or genomic predisposition to AF. This is based on experimental data on PITX2 levels and on the resting membrane potential [36].
2. The recurrence of AF is more common in patients with concomitant heart failure than in those without heart failure (defined as elevated BNP, a surrogate marker for heart failure [37]). For patients with heart failure, we hypothesise that catheter ablation is more effective than antiarrhythmic drugs at preventing AF recurrence [38–40].
3. The recurrence of AF is more common in obese patients (BMI ≥ 30). There is experimental and clinical evidence that increased fatty infiltration and activation of adipocytes in the atria cause AF [22, 41–43], while weight reduction reduces recurrent AF in obese patients with AF [44, 45].
4. The recurrence of AF is more common in patients with severe hypertension (defined as left ventricular hypertrophy on echocardiogram or uncontrolled hypertension at baseline (blood pressure ≥ 160/90)) [46].
5. The recurrence of AF is more common in patients with chronic kidney disease (defined as elevated levels of fibroblast growth factor-23 as a surrogate marker for atrial fibrosis [47–49]).
6. The prevalence of AF is associated with exercise intensity, which has been shown to modulate the relationship between physical activity and AF. Exercise load has been shown to correlate with AF incidence through a U-shaped curve [50].
7. The prevalence of AF is associated with height. Preliminary data suggests that the autonomic tone could mediate this relationship. The association between stature, sex, heart rate and AF will be tested.

## Methods

Studies are eligible for inclusion if they include patients with AF, or who were at risk of AF, identified within the health care system. Longitudinal studies (both observational and randomised trials) are eligible to be included for assessment of recurrent AF, stroke, cardiovascular death and worsening of heart failure. Both prospective and retrospective (including case-control) studies are eligible. Longitudinal and cross-sectional studies are eligible for inclusion for assessment of prevalent AF. Studies have to document diagnoses of AF, key patient characteristics and interventions (where given), and provide individual patient datasets in a form suitable for harmonisation. Twelve studies have been identified from the CATCH ME collaborators which meet these criteria. Our rational for taking this approach instead of searching for eligible studies in a systematic review is that ours is a feasible method in which individual patient data can be obtained in a useful way.

### Description of studies

Individual patient data from the 12 studies will be merged into a single combined dataset (see Additional file 1: Supplementary S1). Data relevant to the design and analysis of the studies were collated from ongoing and closed studies from five European countries (United Kingdom, Germany, France, Netherlands and Spain), contributed by the CATCH ME consortium partners (See Table 1). To achieve comparability of the same latent concepts measured in different studies, the original data coding will be retrospectively harmonised using a semi-automated transformation process during which the source data is assessed and recoded to a common format. The data owners have identified a total of 270 variables (179 baseline, 91 follow-up) covering a wide breadth of information (e.g. patient demographics, rhythm history, cardiovascular disease history, medications, study logistics etc.; see Additional file 1: Supplementary S2). During the harmonisation process, variables from the original dataset will be mapped to their respective target variables. Patient records from seven studies will be augmented by biomarker data obtained by analysing plasma samples from these patients. All biomarkers will be quantified centrally in a core lab using quality-controlled, standardised processes. Ethical approval for repurposing the data will be monitored and overseen by the Atrial Fibrillation NETwork (AFNET, DE).

**Table 1:** Overview of studies

Study (location)	N	Study details	Population	Age (years)	Sex (% males)	Major endpoints	FU (lost to FU)
Flec SL – AFNET 3 [18, 51], (Germany)	635	Controlled trial comparing short- and long-term flecainide therapy after cardioversion in patients with persistent AF	AF	18–89	66%	Recurrent AF. MACE events centrally adjudicated and available.	6 months
AFCT (Netherlands)	388	Observational: Retrospective, case-control	SR, AF	23–74	68%	MACE	2–3 years
BBC-AF (United Kingdom)	1632	Registry	SR, AF	20–97	60%	MACCE. Recurrent AF is captured clinically	2 years
FUTURE (Spain)	198	Registry	SR, AF	22–60	85%	Retrospective follow-up (clinical AF recurrence/incidence)	4–5 years
RACE IV (Netherlands)	1370	Controlled trial of “holistic” AF management and usual care, recently detected AF	AF	19–89	66%	MACE, recurrent AF, AF progression	2–3 years
MULTIAF (Netherlands)	70	Observational: Prospective	AF	33–83	77%	Recurrence of persistent AF	6 months
READ-POAF (Netherlands)	79	Observational	SR	42–84	73%	Incident AF, MACCE	3 years
Tissue bank	246	Combined tissue bank	AF	20–85	70%	Tissue characteristics, no specific follow-up	Variable
AFLMU (Germany)	3573	Ongoing registry of AF patients from a large arrhythmia clinic	AF	16–93	69%	Recurrent AF following intervention (Follow-up available for part of the cohort)	ongoing. Median 1 year FU
KORA (Germany)	4279	Population-based survey including digital ECG, biosamples and detailed characterization	SR	25–74	49%	Incidence of death, stroke, and myocardial infarction	Ongoing since enrolment
GIRAFA [52] (Spain)	210	Patients with lone AF	AF	18–70	70%	Retrospective follow-up	Mean 9 years (AF patients)
PVIBCN [53] (Spain)	1088	Cohort of patients undergoing AF ablation	AF	19–79	74%	Ongoing, systematic follow-up for AF recurrence.	1–2 years

*FU* Follow-up, *SR* Sinus rhythm, *AF* Atrial fibrillation, *MACE* Major adverse cardiovascular events, *MACCE* Major adverse cardiovascular and cerebrovascular events

### Candidate variables for inclusion

**Fig. 1:**
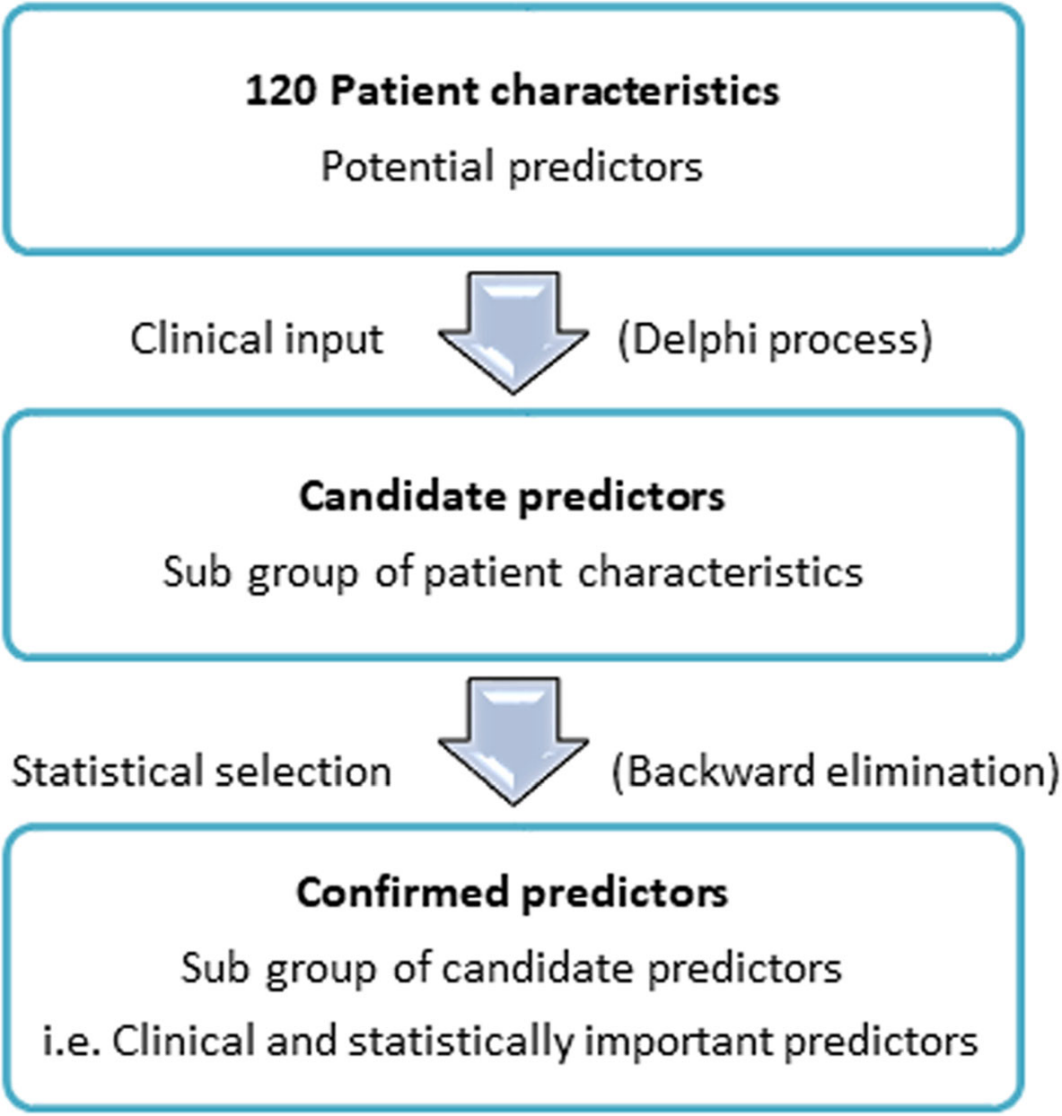
Steps to determine confirmed predictors from a set of patient characteristics that will be used for modelling

One hundred and twenty of the 179 baseline variables are patient characteristics potentially suitable for inclusion in the analysis. The most important patient characteristics will be identified using a Delphi process conducted within the Consortium. These patient characteristics will include variables previously associated with AF as well as markers arising from experiments conducted by CATCH ME partners [22] and others. The Delphi process will rank patient characteristics according to their expected relative importance in predicting recurrent AF and prevalent AF. Similar predictors which assess common clinical concepts will be combined into single variables to minimise the possibility of collinearity within the analysis and lessen the impact of missing data. The highest ranked characteristics will go forward into the statistical selection process (below); the number included will be determined by the sample size calculations (see below) which will establish a subset of characteristics (the *candidate predictors*). In subsequent statistical steps, candidate predictors will be assessed to determine if they are predictive for the outcomes of recurrent AF. This subset of candidate predictors, deemed both clinically and statistically important are referred to as the *confirmed predictors* (Fig. 1).

### Sample size considerations

The number of candidate predictors that can be included in model development is determined by the total number of events for the outcome of interest. We will follow general guidelines that suggest a minimum of 10 events per parameter be considered in a development model [54]. This estimate is known to be conservative for larger lists of parameters, where five events per parameter may suffice [54–57]. Categorical predictors are counted as multiple predictors in this assessment, with the number of indicator variables added to the total count of predictors.

### **Stage 1:** development of predictive models

**Fig. 2:**
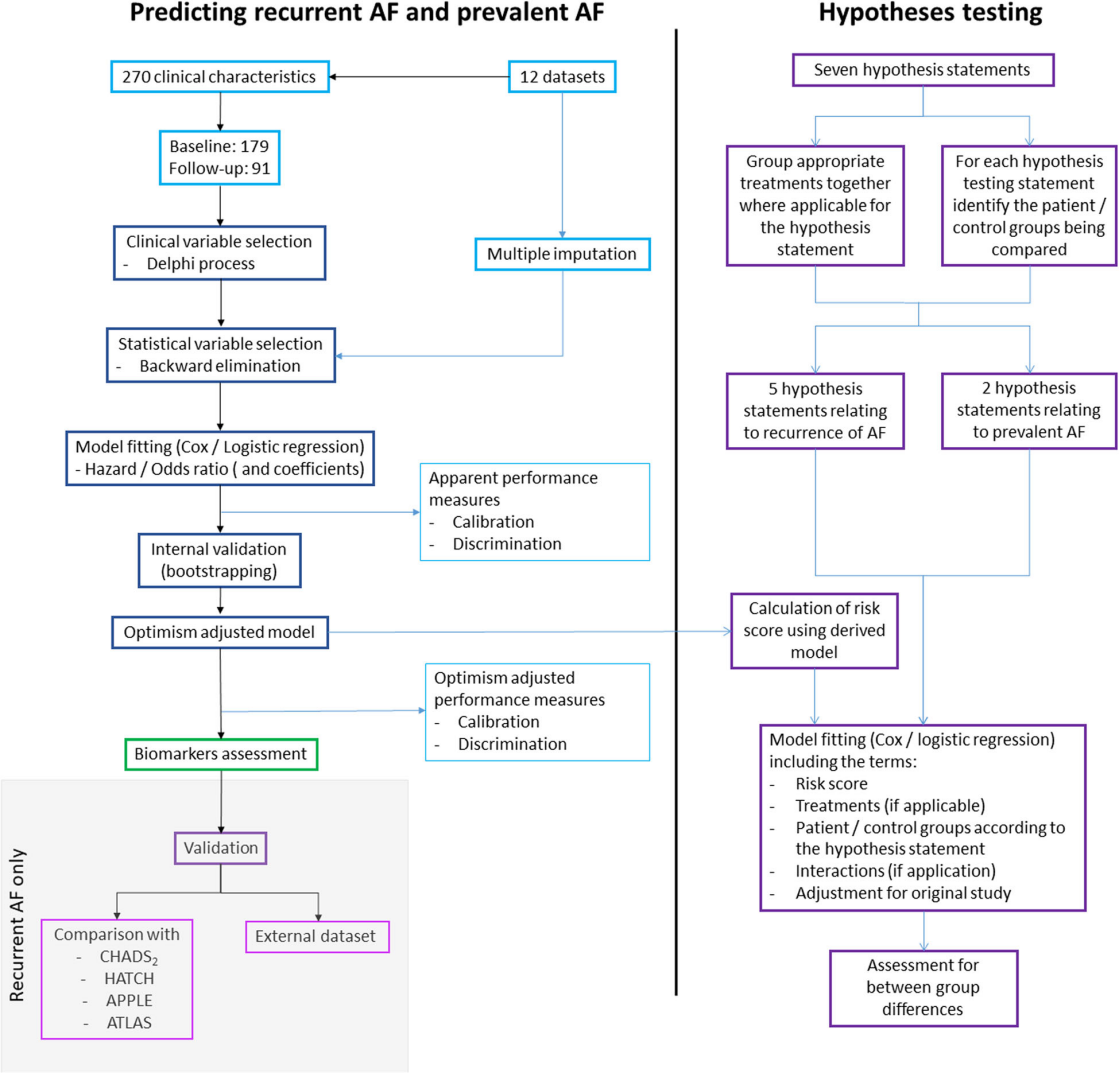
Summary of the analysis on the CATCH ME combined dataset

Cox regression will be used to develop a predictive model for time-to-recurrence of AF in patients previously diagnosed with AF. For other follow-up outcomes (stroke, cardiovascular death, worsening of heart failure), Cox regression models will be used if follow-up time is available, otherwise logistic regression models will be used. Logistic regression will be used for prevalent AF prediction (Fig. 2).

### Missing data

It is expected that the CATCH ME combined database will have missing data for candidate predictors which were not measured in all studies. A detailed assessment of the quantity of missing data across studies and predictor variables will be carried out. We will use multiple imputation using chained equations to account for missing data, assuming data is missing at random (MAR). Multiple imputation will be completed separately for each of the studies after candidate predictors have been identified [58]. Candidate predictors with more than 70% missing (in any study) will be excluded. Characteristics with less than 70% missing data which are not candidate predictors will be included in the multiple imputation procedure as auxiliary characteristics but will not be included as predictors in the models.

The percentage of participants with incomplete data on the candidate predictors will be used to determine the number of imputations required. For example, if in one study 37% of individual participants have incomplete data across all of the eligible candidate predictors then, the number of imputations required would be 37 [59, 60]. Required numbers of imputations will be computed for each study, and we will use the maximum value to ensure it is possible to combine the multiple imputed datasets across all studies.

### Statistical selection and non-linearity

To develop each model we will use backward elimination (BE) to select confirmed predictors from the list of eligible candidate predictors using a *p*-value of 0.157. This significance level has been chosen as it is considered a good proxy to use in place of the Akaike information criterion (AIC) approach [61]. Age and sex will be forced to remain in the statistical selection process irrespective of whether they are statistically significant. The original study will also be included in the model. All continuous predictors will be kept in their original format and will not be dichotomised or categorised to minimise information loss [40]. Continuous characteristics will be assessed for non-linearity. Predictors found to be non-linear will be incorporated using multivariable fractional polynomials (MFP) to allow non-linear relationships. Transforming the continuous predictors using MFP will produce models that are more precise and are more likely to satisfy model assumptions [41].

### Combining multiple imputation and statistical selection procedure

The statistical selection procedure and non-linearity assessments will be performed on the combined imputed datasets (combined across studies and imputations). Multiple imputation datasets are usually combined using Rubin’s rule [40]. However when using statistical selection on multiply imputed data it is likely that for each imputed dataset different predictors will be selected. Hence, the repeated use of Rubin’s Rule across these different selected predictors is computationally challenging. We therefore will use an approximation to Rubin’s rule outlined in Wood, White [62].

### Statistical analysis model and performance measures

Model performance will be assessed, primarily looking at the calibration and discrimination of the model. The calibration of the model will compare the probability of the observed risk to the expected risk using a calibration plot. For determining the models discriminatory ability we will assess using the C-statistic (Harrell’s C-statistic) and its corresponding 95% confidence interval [63].

### Internal validation

To assess optimism and overfitting, the bootstrap method will be used to internally validate the development models. One hundred bootstraps will be used on each of the imputed datasets [64, 65]: each of the imputed and bootstrapped datasets will be analysed individually to determine the calibration and C-statistic values and these will be appropriately combined across the imputed datasets to produce a single value of calibration and C-statistic. The calibration value will be used to adjust the coefficients to ‘shrink’ their value (shrinkage factor) hence allowing us to adjust for over-optimism within our development models. Completing internal validation will affect both the calibration and discrimination values in addition to the coefficient values found in the development model, as they will all be adjusted for the over-optimism. The values reported for the final models will be corrected by the shrinkage factor, along with both optimism adjusted and original C-statistic and calibration values.

### Comparison of developed models to currently available models

We will compare predictions from our new models with those of the CHADS_2_ score [31], HATCH score [32], APPLE score [33], and ATLAS score [34] for recurrence of AF within the CATCH ME combined dataset. Models will be compared using C-statistics.

### **Stage 2:** tests of seven specified hypotheses

The seven a priori hypotheses outlined above will be tested, adjusting for differences in case-mix using the prediction models developed for prevalent and recurrent AF. Cox-regression models will be used for recurrent AF statements and logistic regression for prevalent AF statements. Each model will include a calculation of the predictive risk for each patient computed using the prediction model as a fixed offset term, and additional terms for study to allow for baseline differences in risk. The hypotheses will be tested by adding covariates and interactions to these models. Statements 1–5 pertaining to recurrent AF will use the predicted risk of AF obtained from the predictive model as an offset term. The remaining statements relate to prevalent AF and will use the predicted risk of AF in the same way.

Specifically the first and second hypotheses statements will be assessed by testing for an interaction between treatment and patient status (genetic predisposition for AF/heart failure). Further hypotheses will compare outcomes between two or more groups. These hypothesis tests will be performed on complete data for treatment and these covariates. In summary, the models addressing each hypothesis statement will consist of the predictive risk of AF, the appropriate characteristics and interactions as per the hypothesis and will also adjust for study.

### **Stage 3:** plasma biomarkers

The statistical predictive ability of selected biomarkers quantified in a subset of patients will be investigated for recurrent AF and prevalent AF. The predictive value of each of the biomarkers will be assessed univariately and in addition to the predicted risk of AF recurrence and prevalent AF obtained from the developed models (included as an offset term to adjust for differences in case-mix) whilst adjusting for study. Biomarkers will be considered individually and in combination (see Additional file 2: Supplementary Statistical Analysis Plan for full details).

## Discussion

AF requires chronic and multidimensional management. Providing all treatment options is challenging and ineffective for optimal patient care. While stroke prevention therapy can be selected based on a few clinical parameters in most patients with AF, selecting rhythm control therapy is currently based on personal preferences, local protocols, or availability of treatments. Evidence supporting stratification of rhythm control therapy options in patients with AF would thus meet a clinical need. Similarly, knowledge of the major drivers of recurrent AF could inform strategies to prevent AF, e.g. through targeted modification of risk factors. This retrospective, harmonised, individual patient data analysis will use information from 12 datasets collected in five European countries. Both expert consensus (using a Delphi process) and statistical selection will be applied for a rigorous identification of relevant predictors. Our novel approach will combine clinical characteristics with plasma biomarkers to identify the best predictors of recurrent AF and prevalent AF and use the models to test mechanistic hypotheses and investigate the predictive value of the biomarkers. The heterogeneity of the data will pose analytical and interpretive challenges, however, it will simultaneously drive the pioneering of novel methods for interrogating existing datasets while maintaining the quality and integrity of the data. It is envisioned that the outcome of this analysis would provide a greater understanding of the factors associated with recurrence of AF, potentially allowing development of stratified approaches to prevention of recurrent AF.

## Additional files

Additional file 1: Supplementary S1: Individual dataset descriptions. Supplementary S2: Variables extracted from datasets for the combined database (DOCX 75 kb)

Additional file 2: Statistical analysis plan – CATCH ME –Prognostic development models (DOCX 281 kb)

## Abbreviations

AF: Atrial fibrillation
