# Supplementary material for: Development and external validation of predictive models for prevalent and recurrent atrial fibrillation: a protocol for the analysis of the CATCH ME combined dataset
Source: BMC Cardiovasc Disord. 2019 May 21;19:120. doi: 10.1186/s12872-019-1105-4 (PMC6528378; doi:10.1186/s12872-019-1105-4)
Supplement: Supplementary file 1 — Supplementary S1: Individual dataset descriptions. Supplementary S2: Variables extracted from datasets for the combined database (DOCX 75 kb) [file 12872_2019_1105_MOESM1_ESM.docx]

**SUPPLEMENTARY S1: Individual dataset descriptions.**

**Flec-SL (DE)**

The Flec-SL trial (registration number ISRCTN62728742) is a prospective, randomised, open-label, blinded endpoint assessment trial which was conducted between May 2007 and March 2010 at 44 centres in Germany. Patients with persistent AF undergoing planned cardioversion were recruited. After successful cardioversion, patients were assigned to no drug treatment, short-term treatment, or long-term treatment with Flecainide. Follow-up was conducted after 6 months (1, 2). A total of 635 patient records from Flec-SL were included in the combined analysis.

**Maastricht AF-CT (NL)**

The Maastricht AF-CT cohort is a retrospective, single-centre case-control study which included patients from the Maastricht University Medical Center, Netherlands. Data from patients with AF who underwent cardiac computed tomographic angiography (CTA) as part of work-up for pulmonary vein isolation (PVI), or patients with sinus rhythm who underwent cardiovascular screening between January 2008 and March 2011 were included. Follow-up was conducted by checking records from the centre, referring hospitals, and patient’s General Practices between 2015 and 2016. A total of 388 records from Maastricht AF-CT were included in the combined analysis.

**BBC-AF (UK)**

The BBC-AF Registry is a longitudinal, prospective registry which recruited patients with known AF and patients without AF but had concomitant cardiovascular conditions as assessed by the CHA_2_DS_2_-VASc score, between September 2014 and February 2018 at two centres in the UK. Patients with AF were diagnosed with paroxysmal, persistent, long-standing persistent, or permanent AF. Follow-up was conducted after 2 years. A total of 1632 patient records from BBC-AF were included in the combined analysis.

**FUTURE (ES)**

The FUTURE Registry is a single-centre case-control study which included patients from Hospital Clinic de Barcelona, Spain. Consecutive patients with lone AF episodes were recruited during their first visit to the centre between March 2009 and March 2012. Patient controls were volunteers who did not have any other medical condition. Follow-up was retrospectively performed by investigating electronic medical records. A total of 198 patient records from FUTURE were included in the combined analysis.

**RACE 4 (NL)**

RACE-4 is a prospective randomized controlled event-driven trial with blinded endpoint evaluation in patients with recent onset atrial fibrillation. The treatment of patients with atrial fibrillation is often inadequate due to bad adherence to the guidelines. Nevertheless an integrated chronic care program (ICCP) at a specialized AF clinic for patients with AF may be superior to usual care provided by a cardiologist in terms of cardiovascular hospitalizations and cardiovascular mortality. The primary objective of RACE-4 is to show that an integrated chronic care program at a specialized AF clinic reduces cardiovascular hospitalizations and mortality. The secondary objectives contain the cost effectiveness of the ICCP (expressed in quality adjusted life years), guideline adherence, quality of life, patient knowledge and patient compliance in medication.

**MULTI-AF (NL)**

MULTI-AF is a single-centre prospective observational study in consecutive patients that identifies the main predictors of recurrences of AF after electrical cardioversion in patients with early persistent AF. The co-variates tested include clinical characteristics but also genetic information, circulating serum biomarkers and imaging parameters. Deep phenotyping includes among others trans-oesophageal ECG, a dedicated 17-lead ECG and genetic SNP investigations.

**READ-POAF (NL)**

READ POAF is a single centre, observational study in patients without history of AF undergoing open chest surgery. All patients receive an implantable loop recorder for rhythm monitoring during the operation allowing for quantification of episodes of AF after the intervention. Mean follow up is 2 years.

**KORA S4 (DE)**

The Cooperative Health Research in the Region of Augsburg (KORA) Study is a community-based investigation conducted since 1984 to assess cardiovascular conditions and their modifiers. The Survey S4 was conducted from 1999 to 2001 and enrolled 4261 participants. All participants responded to a detailed interview, received a physical examination and a standardized electrocardiogram, and provided blood for biomarker analyses.

**AFLMU (DE)**

The AFLMU Biobank LMU is part of the Arrhythmia Biobank LMU. It is an ongoing registry of patients with AF treated at the University Hospital of Munich and has been established in 2001. Currently, 3573 patients are available for analysis. Whereas all AF patients are eligible for inclusion into AFLMU, recruitment prioritizes individuals with early onset AF (≤60 years at AF diagnosis) and those undergoing catheter ablation for AF. All enrolled participants provide clinical information on AF as well as concomitant conditions, and provide a blood sample for biomarker analyses.

**GIRAFA (ES)**

The GIRAFA Registry is a single centre case-control study which recruited patients with paroxysmal or persistent lone AF in the emergency room at Hospital Clínic de Barcelona between January 2001 and June 2005. Controls were age- and sex- matched healthy volunteers not related to lone AF patients. Follow-up was retrospectively performed by investigating electronic medical records in patients with AF (3). A total of 210 patient records were included in the combined analysis.

**PVIBCN (ES)**

The PVI-BCN registry is an ongoing Registry including all patients who have undergone an AF ablation procedure at Hospital Clínic de Barcelona since October 2002. For this analysis, patients with ≥2 ablations were included only once (at the first procedure). After the ablation procedure, patients were followed at least at 3, 6 and 12 months post-ablation with a 24-hour Holter at our outpatient clinic. A total of 1,088 patient records were included in the combined analysis. Details of the registry have been reported before (4).

**Tissue Bank (NL)**

The atrial tissue bank of the CATCH ME project is a collection of 245 right or left atrial tissue samples received from 5 partner sites of CATCH ME: University Maastricht, University of Birmingham, University Hospital Munich, University Hospital Barcelona, and Sorbonne University Paris. In the tissue samples histological properties have been analysed using light microscopy and gene expression has been studied using mRNA sequencing. The co-variates tested include clinical characteristics but also genetic information.

**SUPPLEMENTARY S2: Variables extracted from datasets for the combined database.**

| **Category** | **Variable Name** | **Item explanation** |
| --- | --- | --- |
| Patient identification | ID_PT | Study specific ID (original) |
|  | ID_STUDY | Study name (original) |
|  | ID_CATCHME | New ID (automatically generated) |
| Tissue type | TIS_RIGHT | Is right atrial tissue available for analysis? |
|  | TIS_LEFT | Is left atrial tissue available for analysis? |
| Rhythm follow-up after surgery | RHY_PO_POAF | Was post-operative AF (POAF) detected in this patient after the indicated surgery? |
| Ethical approval | ETH_HIST_EXAM | Is histological examination of tissue specimens covered by ethical approval? |
|  | ETH_PROT_ANA | Is protein analysis of tissue specimens covered by ethical approval? |
|  | ETH_GENE_ANA | Is mRNA, miRNA and lncRNA analysis of tissue specimens covered by ethical approval? |
|  | ETH_MAT_TRANS | Are there any legal obstacles for shipment of the material? |
|  | ETH_MAT_TRANS_ex | *If yes, please explain.* |
|  | ETH_BLD_BIO_ANA | Is biomarker analysis of blood/plasma/serum covered by ethical approval? |
|  | ETH_BLD_GENE_ANA | Is miRNA profiling of blood/plasma/serum covered by ethical approval? |
|  | ETH_BLD_SNP_ANA | Is SNP analysis covered by ethical approval? |
| Blood specimen | BLD_SER | Is serum available for this patient? |
|  | BLD_SER_R | *(if yes, what reagent is it in?)* |
|  | BLD_EDTA_PLAS | Is EDTA plasma available for this patient? |
|  | BLD_EDTA_PLAS_R | *(if yes, what reagent is it in?)* |
|  | BLD_BC | Is buffy coat available for this patient? |
|  | BLD_DNA | Is DNA available for analysis? |

| **Category** | **Variable Name** | **Item explanation** |
| --- | --- | --- |
| Rhythm history | RHY_Hx_AF_TYPE | Is there a history of AF? |
|  | RHY_Hx_AF_SYMP | *If yes, is AF symptomatic?* |
|  | RHY_EHRA | What is the EHRA/mEHRA scale of AF symptoms (*if using the mEHRA scale, please select IIa/IIb for score of II*)? |
|  | RHY_AF_Ds_ECG | How many months ago since the first diagnosis of AF on ECG? |
|  | RHY_AF_Ds_DEV | How many months ago since first diagnosis of AF with device (pacemaker, ILR) if different from ECG diagnosis? |
|  | RHY_Hx_AF_ACU | Is there a history of AF in the context of acute illness? |
|  | RHY_Hx_AF_POAF | Is there a history of post-operative AF? |
|  | RHY_Hx_CA | Is there a history of catheter ablation? |
|  | RHY_Hx_CV | Is there a history of cardioversion? |
|  | RHY_Hx_RHY_Sx | Is there a history of rhythm surgery? |
|  | RHY_Hx_AF_Sx_type | *If yes, which type of AF surgery had been previously performed?* |
| Biometric data | pHx_AGE | Age |
|  | pHx_SEX | Gender |
|  | pHx_WT | Weight |
|  | pHx_HT | Height |
|  | pHx_RACE | Race |
| *Prior and/or concomitant cardiovascular consitions* | | |
| Stroke or Transient ischaemic attack (TIA) | CVD_STR_Hx | Is there a history of stroke? |
|  | CVD_TIA_Hx | Is there a history of TIA? |
| Bleeding | HEM_Hx_ICH | Is there a history of intracranial bleeding? |
|  | HEM_Hx_MAJ_HEM | Is there a history of other major bleeding? |
| Hypertension | HBP_Hx | Is there a history of hypertension and / or antihypertensive treatment? |
|  | HBP_Ds | What was the time since first diagnosis (years before inclusion)? |
|  | HBP_SBP | What was the systolic blood pressure at time of inclusion? |
|  | HBP_DBP | What was the diastolic blood pressure at time of inclusion? |

| **Category** | **Variable Name** | **Item explanation** |
| --- | --- | --- |
| Valvular heart disease | VHD_MV | Is there mitral valve disease of at least moderate intensity? |
|  | VHD_TV | Is there tricuspid valve disease of at least moderate intensity? |
|  | VHD_AV | Is there aortic valve disease of at least moderate intensity? |
|  | VHD_PULMON | Is there pulmonary valve disease of at least moderate intensity? |
|  | VHD_Hx_INT_AV | Is there a history of intervention(s) for aortic valve disease? |
|  | VHD_Hx_INT_AV_Type | *If yes, was the intervention a repair or replacement (if surgical and/or catheter)?* |
|  | VHD_Hx_INT_MV | Is there a history of intervention(s) for mitral valve disease? |
|  | VHD_Hx_INT_MV_Type | *If yes, was the intervention a repair or replacement (if surgical and/or catheter)?* |
|  | VHD_Hx_INT_OTH | Is there a history of intervention(s) for other valve disease? |
|  | VHD_Hx_INT_OTH_Type | *If yes, was the intervention a repair or replacement (if surgical and/or catheter)?* |
|  | VHD_M | Was murmur present? |
| Heart failure | CHD_HF_Hx | Is there a history of heart failure (defined as HF symptoms or depressed LVEF)? |
|  | CHD_HF_EF_r_p | What is the HF classification according to ejection fraction? |
|  | CHD_HF_NYHA | What is the NYHA class? |
|  | CHD_HF_IM_DEV | Is there any implanted device? |
|  | CHD_HF_DIA_DI | Is there diastolic dysfunction (defined as grade I [abnormal relaxation] or more)? |
| Diabetes | DM_Hx | Is there a history of diabetes (defined as treatment with oral antidiabetics or insulin)? |
|  | DM_TYPE | If yes, which diabetes type? |
|  | DM_HbA1c | What are HBA1c levels (at inclusion)? |
| Myocardial Infarction | CHD_Hx_mL | Is there a history of myocardial infarction? |
|  | CHD_Hx_STENT | Is there a history of stenting? |
|  | CHD_Hx_CABG | Is there a history of CABG? |
| Hospitalisation | HAD_Hx_CV | Is there a history of hospitalisation for any cardiovascular reason (in the year before inclusion)? |

| **Category** | | **Variable Name** | | **Item explanation** |
| --- | --- | --- | --- | --- |
| Family history for AF or inherited cardiac conditions | | fHx_AF | | Is there family history of AF (1st degree relative with AF)? |
|  |  | fHx_CM | | Is there family history of inherited cardiomyopathy? |
|  |  | fHx_Dx_InCM | | Is there any diagnosed inherited cardiomyopathy in the patient (if yes, what type)? |
|  |  | fHx_CH | | Is there family history of inherited channelopathy? |
|  |  | fHx_Dx_InCH | | Is there any diagnosed inherited channelopathy in the patient (if yes, what type)? |
|  |  | Hx_MUTE | | Was a mutation for cardiomyopathy or channelopathy found? |
| COPD | | COPD_Hx_STEDS_INH | | Is there a history of COPD (defined as treatment with inhalers and / or steroids)? |
| Sleep apnea | | SA_Hx_CPAP | | Is there a history of SA (defined as treated with CPAP)? |
| CKD | | CKD_Hx | | Is there a history of chronic kidney disease? |
|  | | CKD_Hx_STA | | *If yes, which stage?* |
| Other diseases | | PH_Hx | | Is there a history of pulmonary hypertension? |
|  | | RHD_Hx | | Is there a history of rheumatic heart disease? |
|  | | THY_Hx | | Is there a history of thyroid disease? |
| Behavioural risk  Physical activity | |  | |  |
|  |  | PAC_REG | | Was there engagement in regular physical activity? |
|  |  | PAC_DUR | | *If yes, what was the duration of training in hours/week*years of practice?* |
|  |  | PAC_TEAM_ET | | Was there engagement in team sports or endurance sports? |
|  |  | PAC_Hx_COMP | | Is there a history of competitive sport? |
| Smoking | | SMO_Hx | | Is there a history of smoking? |
|  | | SMO_Hx_P-Y | | If yes, what is the total pack-years? |
| Alcohol | | ALC_REG_AC | | Is there regular alcohol consumption? |
|  | | ALC_D | | Is alcohol consumed daily? |
|  | | ALC_D_QTY | | What was the quantity consumed daily? |
|  | | ALC_YR | | What is the duration of consumption (how many years)? |
| Drug abuse | | DA_Hx | | Is there a history of drug abuse? |
| Allergies | | ALL_Hx | | Is there a history of allergies? |
|  | | ALL_Hx_Type | | *If yes, which allergies?* |
| **Category** | **Variable Name** | | **Item explanation** | |
| Medication at inclusion | MED_ASA | | Was the patient on Aspirin at inclusion? | |
|  | MED_VKA | | Was the patient on Vitamin K antagonists (VKAs) at inclusion? | |
|  | MED_NOAC | | Was the patient on Non-vitamin K antagonist oral anticoagulants (NOACs) at inclusion? | |
|  | MED_TIC | | Was the patient on P2Y12 blockers (Ticagrelor, Clopidogrel, Prasugrel) at inclusion ? | |
|  | MED_AD | | Was the patient on Amiodarone at inclusion? | |
|  | MED_DRO | | Was the patient on Dronedarone at inclusion? | |
|  | MED_FLEC | | Was the patient on Flecainide at inclusion? | |
|  | MED_PROP | | Was the patient on Propafenone at inclusion? | |
|  | MED_SOTA | | Was the patient on Sotalol at inclusion? | |
|  | MED_CCBs | | Was the patient on Ca2+ antagonists (any phenylalkylamins, 1,4-dihydrpyridins, or benzothiazepins) at inclusion? | |
|  | MED_ACE | | Was the patient on ACE-inhibitors at inclusion? | |
|  | MED_ARB | | Was the patient on Angiotensin II - receptor blockers (ARBs) at inclusion? | |
|  | MED_BB | | Was the patient on beta-blockers at inclusion? | |
|  | MED_CG | | Was the patient on cardiac glycosides at inclusion? | |
|  | MED_ALDO | | Was the patient on Aldosterone-antagonists at inclusion? | |
|  | MED_SIM | | Was the patient on Statins at inclusion? | |
|  | MED_NSAID | | Was the patient on Nonsteroidal anti-inflammatory drugs (NSAIDs) at inclusion? | |
|  | MED_OGC | | Was the patient on oral glycocorticoides at inclusion? | |
|  | MED_FBR | | Was the patient on fibrates at inclusion? | |
|  | MED_OHA | | Was the patient on oral antidiabetics at inclusion? | |
|  | MED_IN | | Was the patient on insulin at inclusion? | |
|  | MED_DIU | | Was the patient on diuretics other than Aldosterone-antagonists at inclusion? | |
|  | MED_OTH | | Please state if the patient was on any other medication(s) at inclusion. | |

| **Category** | **Variable Name** | **Item explanation** |
| --- | --- | --- |
| ECG | ECG_HR | What is the heart rate? |
|  | ECG_PR | What is the PR interval? |
|  | ECG_QT | What is the QT interval? |
|  | ECG_QTc | What is the QTc interval (Bazett correction)? |
|  | ECG_QRS | What is the QRS interval? |
|  | ECG_PWD | What is the P wave duration? |
|  | ECG_OMI | Are there signs of old infarction on ECG? |
|  | ECG_LVH | Are there signs of left ventricular hypertrophy (LVH) on ECG? |
|  | ECG_IS | Are there signs of acute ischemia on ECG? |
|  | ECG_AEB | Were any atrial ectopics detected on ECG? |
|  | ECG_DIG | Is the digital ECG available (at least 10s, 250Hz sampling rate, 12 bit resolution)? |
|  | ECG_HM | Is the baseline Holter recording available? |
| Cardiac imaging: Echocardiography | ECHO_LAV | What is the left atrial (LA) volume? |
|  | ECHO_LAS | What is the left atrial antero-posterior diameter? |
|  | ECHO_LVF | What is the left atrial (LA) flow? |
|  | ECHO_LVEDV | What is the left ventricular end-diastolic volume (LVEDV)? |
|  | ECHO_LVESV | What is the left ventricular end-systolic volume (LVESV)? |
|  | ECHO_LVEDD | What is the left ventricular end diastolic diameter (LVEDD)? |
|  | ECHO_LVESD | What is the left ventricular end systolic diameter (LVESD)? |
|  | ECHO_EF | What is the ejection fraction? |
|  | ECHO_DSWt | What is the diastolic septal wall thickness? |
|  | ECHO_RAH | Is the right atrium (RA) enlarged? |
|  | ECHO_RAF | What is the right atrial (RA) volume? |

| **Category** | **Variable Name** | **Item explanation** |
| --- | --- | --- |
| Lab values | LAB_INR_i | What is the INR value (time point 1)? |
|  | LAB_INR_ii | What is the INR value (time point 2)? |
|  | LAB_INR_iii | What is the INR value (time point 3)? |
|  | LAB_D-dimer | What is the D-dimer test score? |
|  | LAB_CR | What is the creatinine level? |
|  | LAB_TnI | What is the Troponin I level? |
|  | LAB_TnT | What is the Troponin T level? |
|  | LAB_GLU | What is the glucose level? |
|  | LAB_NT-proBNP | What is the NT-proBNP level? |
|  | LAB_BNP | What is the BNP level? |
|  | LAB_TChol | What is the total cholesterol level? |
|  | LAB_LDL | What is the LDL level? |
|  | LAB_HDL | What is the HDL level? |
|  | LAB_TG | What are the triglycerides levels? |

| **Category** | **Variable Name** | **Item explanation** |
| --- | --- | --- |
| Follow-up | FU_Death | Has the patient died? |
|  | FU_Death_TTE | *if yes, report time from inclusion to death* |
|  | FU_Death_CSR | *if no, report time from inclusion to final follow-up* |
|  | FU_IsSTR | Did the patient have an ischemic stroke? |
|  | FU_IsSTR_TTE | *if yes, report time from inclusion to first ischemic stroke* |
|  | FU_IsSTR_CSR | *if no, report time from inclusion to final follow-up* |
|  | FU_TIA | Did the patient have a TIA? |
|  | FU_TIA_TTE | *if yes, report time from inclusion to first TIA* |
|  | FU_TIA_CSR | *if no, report time from inclusion to final follow-up* |
|  | FU_HeSTR | Did the patient have a hemorrhagic stroke? |
|  | FU_HeSTR_TTE | *if yes, report time from inclusion to first hemorrhagic (intraparenchymal) stroke* |
|  | FU_HeSTR_CSR | *if no, report time from inclusion to final follow-up* |
|  | FU_ICH | Did the patient have any other intracranial bleed? |
|  | FU_ICH_TTE | *if yes, report time from inclusion to first intracranial bleeding (except for intraparenchymal bleeding)* |
|  | FU_ICH_CSR | *if no, report time from inclusion to final follow-up* |
|  | FU_MAJ_HEM | Did the patient have any other major bleeding episodes? |
|  | FU_MAJ_HEM_TTE | *if yes, report time from inclusion to first major bleeding (excluding intracranial)* |
|  | FU_MAJ_HEM_TYPE | Which type of major bleed? |
|  | FU_MAJ_HEM_CSR | *if no, report time from inclusion to final follow-up* |
|  | FU_AF | Did the patient have AF recurrence / receive a de novo diagnosis of AF? |
|  | FU_AF_TTE | *if yes, report time from inclusion to first AF recurrence/new diagnosis* |
|  | FU_AF_CSR | *if no, report time from inclusion to final follow-up* |
|  | FU_AF_Abl | Did the patient have an AF ablation? |
|  | FU_AF_Abl_TTE | *if yes, report time from inclusion to first AF ablation* |
|  | FU_AF_Abl_CSR | *if no, report time from inclusion to final follow-up* |
|  | FU_WT | Weight |

| **Category** | **Variable Name** | **Item explanation** |
| --- | --- | --- |
| Cardiovascular admission | FU_CVadm | Was the patient admitted for any cardiovascular condition? |
|  | FU_CVadm_TTE | *if yes, report time from inclusion to first cardiovascular admission* |
|  | FU_CVadm_CSR | *if no, report time from inclusion to final follow-up* |
|  | FU_CVadm_HF | Was the patient admitted for heart failure? |
|  | FU_CVadm_HF_TTE | *if yes, report time from inclusion to first admission for heart failure* |
|  | FU_CVadm_HF_CSR | *if no, report time from inclusion to final follow-up* |
|  | FU_CVadm_ACS | Was the patient admitted for acute coronary syndrome? |
|  | FU_Cvadm_ACS_TTE | *if yes, report time from incllusion to first admission for an acute coronary syndrome* |
|  | FU_CVadm_ACS_CSR | *if no, report time from inclusion to final follow-up* |
|  | FU_CVadm_Syn | Was the patient admitted for syncope? |
|  | FU_CVadm_Syn_TTE | *if yes, report time from inclusion to first admission for syncope* |
|  | FU_CVadm_Syn_CSR | *if no, report time from inclusion to final follow-up* |
|  | FU_CVadm_PE | Was the patient admitted for peripheral embolism? |
|  | FU_CVadm_PE_TTE | *if yes, report time from inclusion to first admission for peripheral embolism* |
|  | FU_CVadm_PE_CSR | *if no, report time from inclusion to final follow-up* |
|  | FU_ReAF | Was the patient admitted for recurrent AF? |
|  | FU_ReAF_TTE | *if yes, report time from inclusion to first admission for AF recurrence* |
|  | FU_ReAF_Thx | What treatment did the patient receive for AF during admission? |
|  | FU_ReAF_CSR | *if no, report time from inclusion to final follow-up* |
|  | FU_CVadm_OTH | Was the patient admitted for any cardiovascular condition other than listed above? |
|  | FU_CVadm_OTH_TTE | *if yes, report time from inclusion to first admission for other cardiovascular event* |
|  | FU_CVadm_OTH_CSR | *if no, report time from inclusion to final follow-up* |

| **Category** | **Variable Name** | **Item explanation** |
| --- | --- | --- |
| Medication at follow up (12 months) | FU_MED_ASA_12M | Was the patient on Aspirin at 12 months follow-up? |
|  | FU_MED_VKA_12M | Was the patient on Vitamin K antagonists (VKAs) at 12 months follow-up? |
|  | FU_MED_NOAC_12M | Was the patient on Non-vitamin K antagonist oral anticoagulants (NOACs) at 12 months follow-up? |
|  | FU_MED_TIC_12M | Was the patient on P2Y12 blockers at 12 months follow-up? |
|  | FU_MED_AD_12M | Was the patient on Amiodarone at 12 months follow-up? |
|  | FU_MED_DRO_12M | Was the patient on Dronedarone at 12 months follow-up? |
|  | FU_MED_FLEC_12M | Was the patient on Flecainide at 12 months follow-up? |
|  | FU_MED_PROP_12M | Was the patient on Propafenone at 12 months follow-up? |
|  | FU_MED_SOTA_12M | Was the patient on Sotalol at 12 months follow-up? |
|  | FU_MED_CCBs_12M | Was the patient on Ca2+ antagonists at 12 months follow-up? |
|  | FU_MED_ACE_12M | Was the patient on ACE-inhibitors at 12 months follow-up? |
|  | FU_MED_ARB_12M | Was the patient on Angiotensin II - receptor blockers (ARBs) at 12 months follow-up? |
|  | FU_MED_BB_12M | Was the patient on beta-blockers at 12 months follow-up? |
|  | FU_MED_CG_12M | Was the patient on cardiac glycosides at 12 months follow-up? |
|  | FU_MED_ALDO_12M | Was the patient on Aldosterone-antagonists at 12 months follow-up? |
|  | FU_MED_SIM_12M | Was the patient on statins at 12 months follow-up? |

| **Category** | **Variable Name** | **Item explanation** |
| --- | --- | --- |
| Medication at follow up (final) | FU_MED_last | Was there follow-up on medication at other time points? |
|  | FU_MED_TIME_last | If yes, how many months from baseline (please, report last follow-up)? |
|  | FU_MED_ASA_last | Was the patient on Aspirin at last follow-up? |
|  | FU_MED_VKA_last | Was the patient on Vitamin K antagonists (VKAs) at last follow-up? |
|  | FU_MED_NOAC_last | Was the patient on Non-vitamin K antagonist oral anticoagulants (NOACs) at last follow-up? |
|  | FU_MED_TIC_last | Was the patient on P2Y12 blockers at last follow-up? |
|  | FU_MED_AD_last | Was the patient on Amiodarone at last follow-up? |
|  | FU_MED_DRO_last | Was the patient on Dronedarone at last follow-up? |
|  | FU_MED_FLEC_last | Was the patient on Flecainide at last follow-up? |
|  | FU_MED_PROP_last | Was the patient on Propafenone at last follow-up? |
|  | FU_MED_SOTA_last | Was the patient on Sotalol at last follow-up? |
|  | FU_MED_CCBs_last | Was the patient on Ca2+ antagonists at last follow-up? |
|  | FU_MED_ACE_last | Was the patient on ACE-inhibitors at last follow-up? |
|  | FU_MED_ARB_last | Was the patient on Angiotensin II - receptor blockers (ARBs) at last follow-up? |
|  | FU_MED_BB_last | Was the patient on beta-blockers at last follow-up? |
|  | FU_MED_CG_last | Was the patient on cardiac glycosides at last follow-up? |
|  | FU_MED_ALDO_last | Was the patient on Aldosterone-antagonists at last follow-up? |
|  | FU_MED_SIM_last | Was the patient on statins at last follow-up? |
| Censor details | FU_censor_study | At what date did the study stop following patients up (study censor)? |
|  | FU_lost | Was the patient lost to follow up? |
|  | FU_censor_pt_date | If yes, when was the patient censored? |
|  | FU_time | Total follow-up time in days |

**References**

1. Kirchhof P, Andresen D, Bosch R, Borggrefe M, Meinertz T, Parade U, et al. Short-term versus long-term antiarrhythmic drug treatment after cardioversion of atrial fibrillation (Flec-SL): a prospective, randomised, open-label, blinded endpoint assessment trial. Lancet. 2012;380(9838):238-46.

2. Kirchhof P, Fetsch T, Hanrath P, Meinertz T, Steinbeck G, Lehmacher W, et al. Targeted pharmacological reversal of electrical remodeling after cardioversion--rationale and design of the Flecainide Short-Long (Flec-SL) trial. Am Heart J. 2005;150(5):899.

3. Mont L, Tamborero D, Elosua R, Molina I, Coll-Vinent B, Sitges M, et al. Physical activity, height, and left atrial size are independent risk factors for lone atrial fibrillation in middle-aged healthy individuals. Europace. 2008;10(1):15-20.

4. Berruezo A, Tamborero D, Mont L, Benito B, Tolosana JM, Sitges M, et al. Pre-procedural predictors of atrial fibrillation recurrence after circumferential pulmonary vein ablation. Eur Heart J. 2007;28(7):836-41.
