# Supplementary material for: Development and external validation of predictive models for prevalent and recurrent atrial fibrillation: a protocol for the analysis of the CATCH ME combined dataset
Source: BMC Cardiovasc Disord. 2019 May 21;19:120. doi: 10.1186/s12872-019-1105-4 (PMC6528378; doi:10.1186/s12872-019-1105-4)
Supplement: Supplementary file 2 — Statistical analysis plan – CATCH ME –Prognostic development models (DOCX 281 kb) [file 12872_2019_1105_MOESM2_ESM.docx]

Statistical analysis plan – CATCH ME –Prognostic development models

Table of Contents

[1. Introduction 5](#_Toc4752366)

[2. Background and rationale 5](#_Toc4752367)

[Development of prognostic models (WP3) 6](#_Toc4752368)

[3. Study aims and objectives 6](#_Toc4752369)

[4. Description of study design 6](#_Toc4752370)

[4.1. Study data 6](#_Toc4752371)

[4.1.1. Study 1 – AF-CT 6](#_Toc4752372)

[4.1.2. Study 2 – AFLMU 7](#_Toc4752373)

[4.1.3. Study 3 – BBC-AF 7](#_Toc4752374)

[4.1.4. Study 4 – Flec-SL 7](#_Toc4752375)

[4.1.5. Study 5 - FUTURE 7](#_Toc4752376)

[4.1.6. Study 6 – GIRAFA 7](#_Toc4752377)

[4.1.7. Study 7 – MULTI-AF 7](#_Toc4752378)

[4.1.8. Study 8 – Maastricht tissue bank 7](#_Toc4752379)

[4.1.9. Study 9 – PVIBCN 8](#_Toc4752380)

[4.1.10. Study 10 – RACE4 8](#_Toc4752381)

[4.1.11. Study 11 – READ-POAF 8](#_Toc4752382)

[4.1.12. Study 12 – KORA 8](#_Toc4752383)

[5. Outline of the prediction models 8](#_Toc4752384)

[5.1. Characteristics of Participants 8](#_Toc4752385)

[5.2. Inclusion and exclusion criteria 8](#_Toc4752386)

[5.2.1. Recurrence of Atrial Fibrillation (time to event) 8](#_Toc4752387)

[5.2.2. Prevalent Atrial Fibrillation (cross sectional) 9](#_Toc4752388)

[5.2.3. Secondary outcomes 9](#_Toc4752389)

[5.3. Outcome measures 9](#_Toc4752390)

[5.3.1. Outcomes according to prediction models 9](#_Toc4752391)

[6. Proposed statistical methods 10](#_Toc4752392)

[6.1. Overview of the Statistical Model 11](#_Toc4752393)

[6.2. Prediction models 11](#_Toc4752394)

[Model 1 11](#_Toc4752395)

[Model 2 11](#_Toc4752396)

[Model 3 11](#_Toc4752397)

[6.2.1. Interactions 12](#_Toc4752398)

[6.2.2. Study effects 12](#_Toc4752399)

[6.3. Selection of candidate predictors 12](#_Toc4752400)

[6.3.1. Why use a Delphi process? 12](#_Toc4752401)

[6.3.2. How the Delphi process works 12](#_Toc4752402)

[6.3.3. How the Delphi survey will be conducted 13](#_Toc4752403)

[6.4. Sample size 15](#_Toc4752404)

[6.5. Grouping variables together 15](#_Toc4752405)

[6.6. Statistical selection of prognostic factors 15](#_Toc4752406)

[6.7. Handling missing data 16](#_Toc4752407)

[6.7.1. Classification of missing values 16](#_Toc4752408)

[6.7.2. Multivariate Imputation by Chained Equations (MICE) 16](#_Toc4752409)

[6.7.3. Conditional imputation 16](#_Toc4752410)

[6.7.4. Imputing data for non-linear terms 16](#_Toc4752411)

[6.7.5. Imputing incomplete ratios 17](#_Toc4752412)

[6.7.6. Auxiliary variables 17](#_Toc4752413)

[6.7.7. Multiple study data 17](#_Toc4752414)

[6.7.8. Interactions in the imputation model 17](#_Toc4752415)

[6.7.9. Imputation on Cox-regression models 17](#_Toc4752416)

[6.7.10. Number of imputations 17](#_Toc4752417)

[6.7.11. Combining MI datasets 18](#_Toc4752418)

[6.7.12. Imputation of data alongside model selection and non-linear assessment 18](#_Toc4752419)

[6.7.13. Limitations 18](#_Toc4752420)

[6.7.14. Missing data table 18](#_Toc4752421)

[6.8. Internal validation 18](#_Toc4752422)

[7. Model performance 19](#_Toc4752423)

[7.1.1. Sensitivity and specificity 19](#_Toc4752424)

[7.1.2. Receiver Operating Characteristic (ROC) curve 19](#_Toc4752425)

[7.1.3. Risk cut-off points 19](#_Toc4752426)

[7.2. Sensitivity analysis 19](#_Toc4752427)

[8. Hypothesis testing (Stage 2) 20](#_Toc4752428)

[8.1. Analytical approach 21](#_Toc4752429)

[9. Reporting 21](#_Toc4752430)

[9.1. Baseline characteristics 21](#_Toc4752431)

[9.2. Primary outcomes 21](#_Toc4752432)

[9.3. Secondary outcomes 22](#_Toc4752433)

[9.4. Levels of confidence intervals 22](#_Toc4752434)

[10. Limitations 22](#_Toc4752435)

[External validation of the prognostic models (WP4) 23](#_Toc4752436)

[11. Aims and objectives 23](#_Toc4752437)

[11.1. Methods 23](#_Toc4752438)

[11.2. External validation 23](#_Toc4752439)

[11.3. Model comparisons 24](#_Toc4752440)

[12. Statistical software 24](#_Toc4752441)

[13. References 25](#_Toc4752442)

[14. Appendix 28](#_Toc4752443)

[14.1. Study characteristics table 28](#_Toc4752444)

[14.2. Sample size tables for each outcome 29](#_Toc4752445)

[14.2.1. Prevalent AF 29](#_Toc4752446)

[14.2.2. Recurrence of AF 29](#_Toc4752447)

[14.2.3. Stroke 29](#_Toc4752448)

[14.2.4. Cardiovascular death 29](#_Toc4752449)

[14.2.5. Worsening of heart failure 29](#_Toc4752450)

[14.3. Baseline characteristics table 30](#_Toc4752451)

[14.3.1. Primary outcomes 30](#_Toc4752452)

[14.3.2. Secondary outcomes 31](#_Toc4752453)

[14.4. Primary outcome results 33](#_Toc4752454)

[14.4.1. Prevalent AF 33](#_Toc4752455)

[Recurrence of AF 34](#_Toc4752456)

[14.5. Secondary outcome results 35](#_Toc4752457)

[14.5.1. Stroke 35](#_Toc4752458)

[14.5.2. Cardiovascular death 36](#_Toc4752459)

[14.5.3. Worsening of heart failure 36](#_Toc4752460)

[14.6. Equations for all models produced 37](#_Toc4752461)

[14.7. Model performance tables 38](#_Toc4752462)

[14.7.1. Primary outcomes 38](#_Toc4752463)

[14.7.2. Secondary outcomes 40](#_Toc4752464)

[14.8. Calibration 43](#_Toc4752465)

[14.9. Discriminants 44](#_Toc4752466)

[14.9.1. Receiver operating characteristic curve 44](#_Toc4752467)

[14.9.2. Concordance-Statistic (C-statistic) table 45](#_Toc4752468)

[14.10. Missing data 46](#_Toc4752469)

[14.11. Kaplan-Meier estimators 47](#_Toc4752470)

# Introduction

This document gives a detailed statistical analysis plan for the Characterizing Atrial fibrillation by Translating its Causes into Health Modifiers in the Elderly (CATCH ME) study. It will detail all statistical processes for the development of prognostic models required for this project and will most likely cover all eventualities that could occur. Any deviations from this analysis will be documented in the final report or publication in relation to this study.

# Background and rationale

Atrial fibrillation (AF) is the most common cardiac arrhythmia, with a prevalence of 1.5-2% in European populations. By being a major cause of stroke, dementia, heart failure, and premature death, AF is a major threat to the healthy ageing. The main research in Work Package 3 (WP3) will look to develop risk prediction models for recurrence of AF and prevalent AF. The previous stages for this project, Work Package 1 (WP1) and Work Package 2 (WP2), will look to explore and identify statistically significant biomarkers linked to AF. These biomarkers will feed into the prognostic models where WP3 will determine if they are predictive of AF alongside clinical factors. Prior research into predicting episodes of AF have not considered biomarkers that have been identified in WP1 and WP2.

The main focus for WP3 is to identify prognostic factors within a risk prediction model to determine those participants that have the highest risk of having an episode of AF given they have previously been diagnosed with AF, i.e. a recurrence of AF. In addition a separate risk prediction model will be developed to ascertain factors that identify patients at risk of having an episode of AF given no previous diagnosis; this will be known as prevalent AF. In turn this information will help to inform decisions on therapy for patients to achieve the best outcome possible. Subsequent to this we can explore various groups of patients with respect to their risk of having a recurrence of AF and prevalent AF. Formally we will assess these risk groups of patients using carefully set out hypothesis tests (see Hypothesis testing).

In general, prognostic models can be used to assess the predictive ability of patient characteristics known as prognostic factors. In our case the prognostic factors are defined as patient characteristics that have been determined using clinical and statistical methods to be predictive for prevalent AF or recurrence of AF. Both the prognostic factors and biomarkers will be assessed and quantified in relation to predicting AF when undertaking the analysis for WP3. Following this we need to establish how predictive these prognostic factors and biomarkers are in a different set of patients. This will identify if these models are generalisable and hence if these factors and biomarkers are truly predictive for AF. This is known as external validation of the developed prognostic model and forms the sole objective for WP4 (see External validation of the prognostic models (WP4)).

In summary the overall objectives for WP3 are to (1) Identify candidate predictors for predicting recurrence of AF and prevalent AF, (2) Develop risk prediction models for recurrence of AF and prevalent AF using (a) clinical candidate predictors only and (b) clinical candidate predictors and biomarkers, (3) assess the developed risk prediction models performance at identifying participants at risk, (4) assessing hypothesis statements, and (5) fully stating the statistical models depicting the association between characteristics and a patients AF status. Upon completion of WP3, WP4 will begin using the findings from WP3. That is to externally validate the recurrence of AF model within a separate dataset and assess how well this works in a different population, likewise for the prevalent AF model. This in turn will satisfy the objectives for the CATCH ME project to inform future personalised strategies to prevent and treat AF in Europe.

# Development of prognostic models (WP3)

# Study aims and objectives

The primary aim of this study is to develop and internally validate clinical prediction models that combine prognostic factors and biomarkers to predict AF.

The objectives of WP3 are to:

1. Identify candidate predictors of AF within participants that have not been diagnosed with AF (prevalent AF)
2. Identify candidate predictors of AF reccurence within participants that have previously been diagnosed with AF (recurrent AF).
3. Develop risk prediction models for the primary outcomes of prevalent AF and recurrence of AF. These models will only include clinically confirmed candidate predictors identified through clinical and statistical methods (Stage 1)
4. Test *a priori* hypotheses using the developed prognostic models to adjust for differences in case mix (Stage 2)
5. Assess the added benefit of Biomarkers to the risk prediction models (Stage 3)
6. Assess and quantify the prediction models’ performance..

Achieving these objectives for WP3 will aid the external validation (WP4) of the developed prognostic models to be carried out.

# Description of study design

CATCH ME is a retrospective cohort study. The data used for CATCH ME will be pooled from many different studies into one large dataset. The studies that have been chosen to contribute to the CATCH ME database have all been conducted in the EU in the following countries; UK, Germany, Netherlands, France, and Spain, all looking at a range of associations with AF. These contributing studies mainly looked at patient characteristics associated with recurrence of atrial fibrillation with the exception of some studies looking at both new cases of AF and recurrence of AF. All the data pooled from these contributing studies will be harmonised and combined into one large CATCH ME database such that the pooled data is standardised into the same format.

## Study data

The CATCH ME database will include 12 contributing studies which are available to use in WP3. These studies are AFCT, AFLMU, BBC-AF, Flec-SL, FUTURE, GIRAFA, MULTI-AF, Maastricht tissue bank, PVIBCN, RACE4, READ-POAF and KORA. Additional characteristics of each of the contributing studies used will be documented in the appendix in Tables A1-A6.

### Study 1 – AF-CT

The Maastricht AF-CT dataset consists of a mixture of participants whom have either AF or are in sinus rhythm i.e. do not have AF. In total this dataset contains 388 participants of which 273 participants are in sinus rhythm and 115 participants have AF. Within the AF population 46 participants had a recurrence of AF. There is follow up data for both populations in this dataset (387/388) with a total mean follow up time of 2083 days. For the AF population the mean follow up time is 2073 days and for the sinus rhythm population 2087 days.

### Study 2 – AFLMU

The AFLMU study consists of 3573 participants whom have had previous episodes of AF. Of these participants 295 had a recurrence of AF. There is follow up data available however, this is limited as only 632 out of 3573 participants have this information available in relation to if they had a recurrence of AF or not. The mean follow up time for these participants is 376 days.

### Study 3 – BBC-AF

The Birmingham-Black Country-Atrial Fibrillation (BBC-AF) Registry dataset consists of both AF participants and sinus rhythm participants. In total there are 1630 participants in this dataset of which 710 participants have AF and 920 participants are in sinus rhythm. There is no follow up data for this dataset.

### Study 4 – Flec-SL

The Flec-SL study consists of 635 participants whom have previously had episodes of AF. Of the total number of participants, 442 had a recurrence of AF. Follow up data is only available for 480 participants, with a mean time of 80 days. For those that did not have a recurrence of AF, 193 had a follow up time with a mean time of 158 days, and for the 287 participants with follow up data that had a recurrence of AF had a mean of 28 days.

### Study 5 - FUTURE

The FUTURE study consists of both AF participants and sinus rhythm participants. The total number of participants within this dataset is 198, of which 113 have AF and 85 participants are in sinus rhythm. Within the AF population 62 participants had a recurrence of AF. There are 123 participants with follow up data across both populations within this dataset. For the AF population 93 participants had follow up data with a mean time of 1685 days and for the sinus rhythm population only 30 participants had follow up data with a mean time of 1392 days.

### Study 6 – GIRAFA

The GIRAFA study consists of 210 participants of which 104 do not have a history of AF and 106 have a previous diagnosis of AF. Of these 210 participants, 16 had a recurrence of AF. Follow up data is available for a total of 26 participants of which 16 had a recurrence of AF and 10 did not have a recurrence. For those that had a recurrence the mean time they were followed up for was 1984 days, and for those that did not have a recurrence the mean time was 1825 days.

### Study 7 – MULTI-AF

The MULTI-AF study consists of 70 participants that all have a diagnosis of AF. Fifty participants had a recurrence of AF. This dataset does not have follow up data.

### Study 8 – Maastricht tissue bank

The atrial tissue bank of CATCH ME work package 1 is a collection of 245 right or left atrial tissue samples received from 5 partner sites of CATCH ME: University Maastricht, University of Birmingham, University Hospital Munich, University Hospital Barcelona, and Sorbonne University Paris. In the tissue samples histological properties have been analysed using light microscopy and gene expression has been studied using mRNA sequencing. The co-variates tested include clinical characteristics but also genetic information.

### Study 9 – PVIBCN

The PVIBCN study consists of 1088 participants whom have a previous diagnosis of AF. There were 523 participants that had a recurrence of AF. There is follow up data for 872 participants in this dataset, where 483 participants had a recurrence of AF and 389 did not have a recurrence of AF. The mean follow up time for those that had a recurrence was 332 days and 643 days for those that did not have a recurrence.

### Study 10 – RACE4

The RACE4 study consists of 1332 participants who have had a previous diagnosis of AF. This study does not have follow up data.

### Study 11 – READ-POAF

The READ-POAF study consists of 79 participants that are in sinus rhythm i.e. no history of having AF. Forty-six participants had a new diagnosis of AF; however there is no follow up data for these participants and also those participants that did not have a new diagnosis of AF.

### Study 12 – KORA

The KORA study consists of 4279 participants whom have no previous diagnosis of AF. This study had no follow up data collected.

# Outline of the prediction models

## Characteristics of Participants

The data will be collected from a range of participants due to the different contributing studies being pooled together. Their patient characteristics will be recorded in the CATCH ME database. Candidate predictors (Table 1) will be identified from the patient characteristics that have been collected for the CATCH ME database. The candidate predictors are defined as patient characteristics that have been selected on the basis of clinical opinion and hence identified to be potentially important in predicting the outcome of interest, i.e. prevalent AF and recurrence of AF. A baseline table for these candidate predictors and biomarkers found in WP1 and WP2 for these participants will be produced (See Table A7-A11).

## Inclusion and exclusion criteria

The participants that are entered into the study have to meet a specific criterion to comply with the primary outcomes. There are different criteria for which patient information can be utilised in the prediction models, namely participants will have to display certain characteristics to be considered for the primary outcomes of predicting a recurrence of AF and prevalent AF. This will be addressed at the study level to establish if the original study meets the criteria to be included in their respective analyses for WP3.

### Recurrence of Atrial Fibrillation (time to event)

To be included in this analysis, participants need to be previously diagnosed as having AF, however cannot have permanent AF at the time of entry into the study. Additionally follow up times for this outcome needs to be available.

### Prevalent Atrial Fibrillation (cross sectional)

Contributing studies that are eligible to be used for assessing characteristics and AF status have to contain both participants that have AF and participants that are in sinus rhythm (no previous diagnosis of AF). Follow up times are not required from these studies as this is an assessment at one point in time (baseline).

### Secondary outcomes

Participants that will contribute to the analysis for the secondary outcomes of stroke, cardiovascular death and worsening of heart failure need to be previously diagnosed as having AF. For all of these secondary outcomes, follow up data is required for Cox regression to be conducted.

## Outcome measures

**Diagram 1**: Displaying the flow from the studies that data has been collected from to separating them out according to their population followed by the corresponding primary and secondary outcomes that are applicable to carry out.

### Outcomes according to prediction models

Due to the two different populations defined by their AF status, the prediction models are detailed below in accordance to the relevant outcome (Diagram 1).

#### Primary

1. **Recurrence of AF:** A population of participants that have previously been diagnosed with AF at baseline and could go on to have a recurrence of AF in the future.
2. **Prevalent AF:** A population of participants that either have previously been diagnosed with AF or no history of AF is required to assess characteristics in relation to AF status within this population at one point in time.

#### Secondary

1. **Stroke:** A population of participants that have previously been diagnosed with AF at baseline and could go on to have a stroke in the future.
2. **Cardiovascular death:** A population of participants that have previously been diagnosed with AF at baseline and could go on to have a cardiovascular death in the future.
3. **Worsening of heart failure:** A population of participants that have previously been diagnosed with AF at baseline and could progressively have a worsening of heart failure in the future.

| Terms | Definitions |
| --- | --- |
|  |  |
| Patient characteristics | Any characteristic a patient or participant has that is documented in the CATCH ME database. |
|  |  |
| Candidate predictor | A subset of the patient characteristics that have been selected clinically via a suitable process (e.g. literature review, Delphi survey) deemed to be potentially associated with the disease or outcome. |
|  |  |
| Prognostic factors/confirmed clinical predictors | A subset of the candidate predictors selected using another suitable process (e.g. Statistical selection) are therefore used in a prediction model. |
| Prognostic index | The linear predictor value without the inclusion of study |
| Covariate | A variable that is used in statistical model. |
|  |  |
| Participant | A participant is someone that has been invited to partake in a process, for example, someone who has been invited to take part in completing a Delphi survey is a participant or someone who has been invited to take part in a study. |
|  |  |
| Prognostic model/  Risk prediction model/  Prediction model | A model predicting an outcome (e.g. Atrial fibrillation) using a set of prognostic factors (e.g. Age, gender, ethnicity,…). |
|  |  |
| Worsening of heart failure | Defined as the patient having one of the following characteristics:   1. Progressing to a higher NYHA classification at follow up compared to baseline, e.g. NYHA classification of II at baseline and at follow up the patient has a NYHA classification of IV would indicate a worsening of heart failure. 2. progressing from a higher percentage of ejection fraction level to a lower level at follow up, i.e. from >50% at baseline to <50% at follow up 3. Having a greater number of hospital admissions for cardiovascular reasons at follow up compared to baseline. |

**Table 1:** Table of definitions

# Proposed statistical methods

Many steps are required to develop these prediction models, including the clinical selection of candidate predictors, statistical selection of prognostic factors, imputation on missing data, use of non-linear methods, statistical analysis, internal validation and assessment of model performance i.e. whether the model is able to make good predictions in the developmental dataset. In this section we will address each step and how it will aid the development of the prediction models that we propose to undertake.

## Overview of the Statistical Model

The primary aims of this study are to predict prevalent AF and recurrence of AF. The statistical analysis that will be performed are logistic and Cox regression (proportional hazards regression), respectively. When developing the model for recurrence of AF, Cox regression will be used and thus uses outcome data in the form of time values with an indication of if the patient has a recurrence of AF. An event is defined as having the outcome of interest, however if a patient does not have an event then the patient will be censored. This means that the time recorded for a patient that does not have an event within the study will have a time that indicates the last time they were followed up in the original study. Participants can also withdraw from the original study for various reasons and therefore they will be assigned a time corresponding to the last follow up time known to the original study where they did not have an event. These participants that withdrew are known to be ‘lost to follow up’, i.e. they did not remain in the study for the whole duration.

When developing the prevalent AF model, logistic regression will be used and therefore the outcome will have been recored in binary form, i.e. if they have ever had a diagnosis of AF or not.

For the secondary outcomes: worsening of heart failure, cardiovascular death and stroke, they will again be recorded as time values with an indication of if the patient has a worsening of heart failure, a cardiovascular death or a stroke, respectively. This is due to again using cox regression to model these outcomes as these patients all have a diagnosis of AF.

If insufficient time to event data is recorded for any of the primary and secondary outcomes across the appropriate studies, then another regression will be used for the statistical analysis to take advantage of the data that has been collected, for example using logistic or Poisson regression.

## Prediction models

This study will consist of 3 main prediction models per outcome:

- **Model 1:** Prognostic factors (Stage 1) (see Table 1 and 2 for definitions).
- **Model 2:** Hypothesis driven models adjusting for prognostic factors (Stage 2)
- **Model 3:** Biomarkers adjusting for prognostic factors (Stage 3).

### Model 1

The prognostic factors included in this model are identified by the clinical selection process (see Selection of candidate predictors) and statistical selection process (See Statistical selection of prognostic factors). Model 1 will assess and quantify the effect of these prognostic factors from the demographic (age and gender) and clinical predictors only for each of the outcomes.

Model 2 – See Section ‘Hypothesis testing (Stage 2)’

### Model 3

The prognostic factors combined with the biomarkers will be used in this model. The addition of the biomarkers to the prognostic factors will help to understand and quantify the potential predictive added value of the biomarker over that of the prognostic factors identified, for each of the two prognostic models.

### Interactions

The models will look into an age and gender interaction as this is known to be associated with the primary outcomes (Heeringa et al., 2006). No other interactions will be investigated.

### Study effects

Due to combining different study data, this needs to be included and adjusted for in every model. Each study data will differ from one another due to varying populations from an array of countries they were conducted in. Therefore the study will be accounted for in all models to allow for such differences, whereby they will be included as just another variable within each of the prognostic models.

## Selection of candidate predictors

In the first instance the clinical selection of candidate predictors needs to be undertaken. In this study a Delphi process will be used to clinically select these candidate predictors to take forward to the next stage of the process to develop the prognostic model (Diagram 2).

### Why use a Delphi process?

Identifying predictors to be included in a prognostic model is itself an important task. The naive ideal would be that any/all patient characteristics are incorporated into the prognostic model. However, this leads to multiple problems, such as overfitting, which leads to non-generalisable prognostic models for different populations. Therefore identifying the most important and relevant patient characteristics for the specific disease is very important if the model is to be used in practice. Candidate predictors are often selected in a non-systematic way, likely to be informed in some way from a-priori beliefs as to what predictors are clinically important, but often driven by availability of data. More formal ways of identifying candidate predictors include literature or systematic reviews and expert opinions, although there is currently no formally recommended approach for selecting candidate predictors. Expert opinions might be derived informally or more formally using consensus based methods such as a Delphi process.

### How the Delphi process works

The Delphi process will be utilised in this study to identify the most important patient characteristics in predicting first diagnosis of AF and recurrent AF. These candidate predictors identified by the Delphi process will then be taken forward to the statistical analysis.

One hundred and twenty potential patient characteristics were available for consideration in the selection process to be candidate predictors. These patient characteristics are linked to AF with varying strengths of association and therefore will be used in a Delphi survey. Experts in Cardiology from the CATCH ME consortium will form the expert panel of whom will be invited to complete the Delphi survey. The Delphi survey will be completed by each of these participants from the expert panel. Once completed, the survey results will be analysed to assess if a general consensus for each of the patient characteristics has been achieved. If a general consensus has been reached for all patient characteristics then another survey is not required.

However if no general consensus has been reached across the patient characteristics i.e. at least one of the characteristics has not gained a consensus, then a second round of the Delphi survey is required. This second round will contain just the patient characteristics that did not gain a consensus. More rounds of the survey will be required to be completed until all patient characteristics have gained a general consensus. Each new round of the Delphi survey will additionally include results (from the previous round) of the patient characteristic which did not gain consensus from the previous round. Thus giving the participants an insight in to how other participants rated that specific characteristic on its predictive ability in relation to the primary outcome. By informing the participant of others opinions will, in theory, converge the results to gain a general consensus on all remaining patient characteristics. In the case that there are outstanding characteristics that have not reached consensus after a number of rounds of the Delphi survey, a meeting will be arranged to achieve consensus on the remaining patient characteristics.

### How the Delphi survey will be conducted

The participants completing the survey will rate each patient characteristic (independent of any other characteristic) according to how important it is in predicting a first diagnosis of AF or a recurrence of AF. These patient characteristics will be rated using a 6-point Likert scaling system.

The Delphi survey will be constructed such that the patient characteristics are grouped appropriately. This survey will consist of the 120 patient characteristics available to us, linked to AF. Firstly, age, gender and ‘contributing study’ will be grouped together and from now on will be known as Demographic predictors (Table 2). However, ‘contributing study’ will not be included in the Delphi survey. The remaining baseline clinical characteristics (n=118) will be grouped in the following way in the Delphi survey:

- Family history (AF/channelopathy/cardiomyopathy)
- Lifestyle factors (physical activity, smoking, alcohol, drug abuse)
- Rhythm follow-up after surgery
- Rhythm history
- Cardiovascular disease
- Haemorrhage
- Hypertension
- Valvular heart disease
- Heart failure
- Diabetes
- Co-morbidities (Chronic obstructive pulmonary disease, Sleep apnea, Chronic kidney disease, allergies, other diseases)
- Medication at inclusion
- Electrocardiogram (ECG)
- Cardiac imaging: Echocardiography

Once the Delphi survey has been completed this will provide us with a complete list of all the patient characteristics and how the participants rated each of them with respect to their importance of predicting the two primary outcomes. The patient characteristics will be ordered from most important to least important in predicting the primary outcomes with respect to their mean scores. According to the sample size calculations (see Sample size) to determine how many candidate predictors can be included in the next stage of developing the prognostic model, we can apply a cut-off value to this ordered list of patient characteristic. The patient characteristics that are the most important, identified after the cut-off value has been applied, will be referred to as clinical predictors which do not include the demographic predictors (Table 2).

The third group of potential predictors are biomarkers identified in work package 1 (WP1) and work package 2 (WP2) of this study. The biomarkers found from these work packages will be referred to as biomarkers and will not be included in the Delphi survey.

Clinical input (i.e. Delphi process)

Statistical selection (i.e. Backward elimination)

**Diagram 2:** Flow diagram for the selection of prognostic factors from patient characteristics in WP3.

| Classification of potential predictors | Definition |
| --- | --- |
| Demographic predictors | This set of patient characteristics will only including age, gender and ‘contributing study’. |
|  |  |
| Clinical predictors | This set of patient characteristics include the most important characteristics found using the Delphi selection process in conjunction with the cut-off value (found using the sample size calculation) to gain a subset of patient characteristics. |
| Clinical candidate predictors | This set of patient characteristics includes the demographic predictors and clinical predictors only. |
|  |  |
| Biomarkers | This group contains the biomarkers identified as significant in predicting AF from WP1 and WP2 of this study. |

**Table 2:** The definitions of the classification of potential predictors. These potential predictors are also identified as candidate predictors if used in context before the statistical selection has occurred.

## Sample size

The number of candidate predictors that can be included in the development model relates to the total number of events for the outcome of interest. General guidelines suggest a minimum of 10 events per parameter considered in the development model (Peduzzi et al., 1996). This in general indicates that we need about 10 events per candidate predictor for inclusion to the statistical selection process. However, for categorical predictors with more than two categories to select from would also need to be taken into account when considering the guidelines, as they would count towards the parameter total. The number of events will inform on how many of the highly important candidate predictors (found using the Delphi process) will be used in the statistical selection of prognostic factors, taking into account the categorical candidate predictors. A template table to gather the relevant information per outcome, per study has been documented in the Appendix (Table A2-A6).

## Grouping variables together

As there are 120 patient characteristics previously identified by the project consortium linked to atrial fibrillation, inevitably there will be a large amount of overlap in these characteristics and hence could indicate the same clinical aspect. For example many different antiarrhythmic drugs are documented as individual variables when in fact they could be grouped together. By grouping these individual variables together a separate indicator variable will be created, therefore, any patient taking any of these specific antiarrhythmic drugs will have a data entry in this new indicator variable named ‘antiarrhythmic drugs’.

By grouping these variables together allows for consolidation of variables and therefore the newly created variable could be a candidate predictor in place of the multiple specific variables. These groupings of variables will be discussed at a clinical level with selected members of the consortium. Additionally by grouping these variables this will take advantage of the data that is available from each of the studies that are relevant for each outcome.

## Statistical selection of prognostic factors

When statistically selecting the clinical candidate predictors for inclusion into each of the models for the primary and secondary outcomes, a selection process will be used. To develop each model we will use backward elimination (BE) to select confirmed predictors from the list of eligible candidate predictors using a p-value of 0.157, i.e. a variable is dropped if its removal causes a non significant increase in deviance (Wood et al., 2008, Morris et al., 2015).This significance level has been chosen as it is considered a good proxy to use in place of the Akaike information criterion (AIC) approach (Sauerbrei, 1999). Age and sex will be forced to remain in the statistical selection process irrespective of whether they are statistically significant. The original study will be included as a fixed effect.

When conducting the statistical variable selection, the demographic predictors will be ‘forced’ to remain in each of the models proposed. The clinical predictors will be included in the statistical variable selection in addition to the demographic predictors. The candidate predictors that are statistically significant and meet the criteria as defined above will remain in the model. This first model will be the base model which will be referred to as Model 1 (as described previously). This model will hence contain the demographic and clinical prognostic factors that are clinically and statistically identified to be prognostic to the outcome.

Model 3 will build upon model 1 where by the biomarkers will be added to the prognostic factors found in the statistical selection process for model 1. Specifically, the prognostic index obtained from model 1 for each patient will be used in a separate model (model 3) as an offset term. In addition to this offset term the biomarkers will be added, both singularly and in combination. The outcome for model 3 will remain the same as in model 1.

## Handling missing data

In the first instance, the data will be rigorously collected for each study in an appropriate manner to minimise the amount of missing data for the CATCH ME database. If there is missing data this will be dealt with using multiple imputation using chained equations.

### Classification of missing values

The missing values in our dataset will be assumed to be Missing At Random (MAR) that is the values are missing dependent on the observed data and not on the missing data itself. Imputing these missing values requires an imputation model containing the covariates (complete and incomplete covariates) that will be included in the statistical model, along with the outcome variable. If the outcome is not included in the imputation the covariate outcome association will be underestimated.

### Multivariate Imputation by Chained Equations (MICE)

Multivariate imputation is conducted using a process called Multivariate Imputation by Chained Equations (MICE). Simply we aim to get a ‘complete’ multivariate sample of our data which equates to one imputed dataset. This is achieved using the covariates that will be included in the model that have already been identified prior to imputation, along with the outcome variable with the addition of auxiliary variables (see section 8.7.6). The continuous covariates included in the imputation model must be assumed to be normal. If these continuous covariates are not normally distributed then they need to be transformed for the assumption to hold when carrying out multiple imputation.

### Conditional imputation

Within our dataset there are variables that are conditional i.e. a variable depends on another variables’ answer. For example we could have a variable ‘Does the patient smoke?’ with possible answers ‘Yes’, ‘No-never’ and ‘No – ex-smoker’. Following on from this variable we could have the variable ‘If yes, how many times do you smoke a day?’ with the answers ‘0-10’, ‘11-20’, ‘21-30’, ‘31-40’ and ‘41+’. This structure of questions could cause problems when imputing missing values as a patient that smokes (indicated by the first variable) many not specify the amount of times they smoke a day. Also if the first variable answer is missing then consequently the second variable would also be missing as it depends on the first variables’ answer. This can be accounted for and addressed by specifying this structure within the programming of the imputation within STATA.

### Imputing data for non-linear terms

Non-normal missing data needs to be addressed to satisfy the normality assumptions for MAR data and multiple imputation. If a continuous variable looks to be non-normal and there are missing values that need to be imputed then we need to address this by transforming them prior to imputation.

Additionally, Seaman et al. (2012) looked into the best approaches in handling non-linear terms when considering multiple imputation. They concluded that no particular method performed better than any other. However having a non-linear variable included in the multiple imputation was best utilised by treating it as ‘just another variable’ i.e. imputing X and X^2^ separately ignoring the relationship between them.

### Imputing incomplete ratios

Analyses including such variables as BMI sometimes contain variables behind them i.e. BMI uses variables height and weight. To account for these ratios especially if there are missing values in height, weight and BMI we need to know how to best approach this as to not bias the results. In this instance we will be imputing missing values is to impute height, weight and BMI separately as to treat BMI as ‘just another variable’ (Morris et al., 2014).

### Auxiliary variables

If a covariate being included in the analysis model depends on other variables, these other ‘background’ variables need to be included in the multiple imputation model. These variables are known as ‘auxiliary’ variables as they are associated with the outcome but do not belong in the analysis model. Including these auxiliary variables in the imputation model in essence reduces standard errors and provides a richer imputation model than the analysis model producing more plausible results from the analysis model. Candidate predictors with more than 70% missing (in any study) will be excluded. Characteristics with less than 70% missing data which are not candidate predictors will be included in the multiple imputation procedure as auxiliary variables but will not be included as predictors in the models.

### Multiple study data

The data from the CATCH ME dataset contains data from many different contributing studies and this needs to be accounted for. This will be addressed by multiple imputing data per dataset as appose to across all of the study datasets. This is due to the varying different inclusion and exclusion criteria for each of the studies (Burgess et al., 2013).

### Interactions in the imputation model

Interactions required or explored in the analysis of the data needs to be accounted for in the imputation model otherwise this could lead to bias in the analysis model and hence the results. This can be simply accounted for and added into the imputation model, enabling the interactions to be accurately represented in the analysis model. We will only be looking at the interaction between age and gender.

### Imputation on Cox-regression models

As a cox-regression model is considered the primary analysis model for the data then this needs to be accounted for in our imputation model needs to be addressed. This can be easily implemented in STATA.

### Number of imputations

The number of imputations that can be used for multiple imputation depends on the amount of missing data. This number should be representative i.e. equate to if an infinite amount of imputations had been carried out, therefore the representative number would be chosen where convergence occurs. This can be found out simply by assessing the proportions of complete and incomplete data entries. For example if 45% of participants had complete data entries for the baseline characteristics and outcome variable then 55 (=100%-45%) imputations would be required.

To determine the number of imputations we require for the multiple imputation we will be assessing the complete/incomplete proportions in each of the datasets separately. The final number of imputations that will be carried out for the multiple imputation of the separate contributing datasets will be the largest number found previously when assessing each of the datasets. This will ensure that when the datasets are pooled together into one large dataset the number of imputations is consistent across each of the separate study datasets.

### Combining MI datasets

Once multiple imputation has been conducted in each of the separate contributing datasets and they have been pooled together into one large dataset we will need to combine the results of the multiple imputations together. Combining these estimates is achieved using Rubin’s rules (Rubin, 2008). This uses combined estimates, mean within-imputation variance, between-imputation variance and total variance to find the parameter values and confidence intervals. In addition the degrees of freedom need to be calculated to aid confidence interval calculations. This does not need to be completed by hand; STATA can complete this combining of values relatively easily.

### Imputation of data alongside model selection and non-linear assessment

Model building is required using the multiple imputed data. This is required as there is no established model and there is also a large amount of data that is likely to have missing data points. This model selection process will look at parameters, interactions and relationships between variables. It is however quite a complex structure on the multiple imputation. This variable selection process is driven by hypothesis tests using Wald tests (H_0_: parameter Q=0, H_1_: parameter Q≠0). The Wald tests are easily obtainable using STATA in the MI analyses. This is a data driven way of building a model. In conjunction with this, fractional polynomials will be assessed at the same time.

Aside from assessing the non-linear effects before conducting multiple imputation in the first instance the continuous prognostic factors need to be assessed to see if they have a linear relationship with the outcome. If they do meet this linearity assumption then they remain in the continuous format unchanged to how they were collected. However, if they display a non-linear relationship to the outcome then they need to be assessed using fractional polynomials. Fractional polynomials are used to establish a non-linear relationship to the outcome and hence will be able to model the data more appropriately. Therefore the overall model will display more accurate relationships and hence clinical and statistical results. This will be used in conjunction with the model selection process to assess for non-linearity relationships.

The statistical selection procedure and non-linearity assessments will be performed on the combined imputed datasets (combined across studies and imputations). Multiple imputation datasets are usually combined using Rubin’s rule (Rubin, 1987). However when using statistical selection on multiply imputed data it is likely that for each imputed dataset different predictors will be selected. Hence, the repeated use of Rubin’s Rule across these different selected predictors is computationally challenging. We therefore will use an approximation to Rubin’s rule outlined in Wood, White (Wood et al., 2008).

### Limitations

Using MICE does come with some limitations when applied to data. Although there is a lack of theoretical basis for MICE, in practice it works relatively well (White et al., 2011).

### Missing data table

A table containing the percentage of missing data from each study for all the candidate predictors is documented in the Appendix (Table A35).

## Internal validation

The final prognostic models will be internally validated using bootstrapping methods. These final models prior to bootstrapping are known as apparent models, and after adjusting for the results of the bootstrapping they are referred to optimism-adjusted models. Bootstrapping will assess the stability of the coefficients produced to prevent over-optimism and over-fitting. This allows the optimism-adjusted model to then be more robust in other datasets and more representative of the population. A presentation of results will be documented before (apparent model) and after internal validation (optimism adjusted model) has taken place to display the adjustment that has been made on the coefficients, calibration and C-statistic.

# Model performance

### Sensitivity and specificity

A template of a table to contain the information about sensitivity and specificity values for each model on each outcome is documented in Tables A22-A31 in the Appendix. This table contains sensitivity and specificity as percentages. Positive predictive values and negative predictive values are also included in the table along with the prevalence of the outcome. The columns in this table are cut-off values that will be used to depict how well the model performs along with a visual representation of these cut-off values in relation to the prediction models.

### Receiver Operating Characteristic (ROC) curve

A receiver operating characteristics (ROC) curve will be created using a clinically determined cut point to establish how well the prognostic models works. In addition the C-statistic will be reported (see example in Figure A1).

### Risk cut-off points

The risk cut-off points need to be established and have clinical meaning to them. These will be established using the clinical input from appropriate CATCH ME partners. This enables more robust and useful cut-off points for clinical practice.

## Sensitivity analysis

Sensitivity analysis tests the robustness of the models that have been developed and to determine how uncertain/certain we are of the models produced. This could include: (1) complete case analysis, (2) how the models perform using practicable clinical predictors and biomarkers, (3) completing the analysis in the separate studies and, (4) only selecting the variables that appear in the hypothesis statements including those variables that are required to be in the model (i.e. age and sex), omitting the need to use statistical selection.

1. Conducting a complete case analysis for each of the models would entail not imputing on the missing data. Templates for the complete case analysis are documented in the appendix (Tables A12-A31).
2. The clinical predictors and biomarkers used as predictors i.e. prognostic factors, for each outcome will not necessarily be used in a practical situation. For example, biomarkers and clinical predictors that are unmeasurable in a consultation room will be included in the developed models for this study. However such prognostic factors will be excluded when conducting the sensitivity analysis. Hence these new models will only contain practicable (measureable) predictors. These new sensitivity check models can be compared to the original models.
3. As this is a retrospective study not all of the collective study data used will be high quality and reliable or even similar to one another. The differences between these studies may impact the selection and magnitude of the variables in the model. Therefore completing the analysis in each of the study datasets will be a good way to assess this and compare back to the original results when the analysis was completed using all study datasets. This would be completed by fitting the prognostic factors found for each risk prediction model to each study dataset.
4. As these hypothesis statements document many variables to be tested in certain sets of patients with the inclusion of certain treatments of interest, it would be a good way to assess all of these characteristics in combination. The variables that have been specifically stated in these statements are of clinical interest and hence including them in the statistical model for their appropriate outcome would be of particular interest. This could allow for variables that were not previously selected into the prognostic model due to the statistical selection process. Therefore by selecting these variables outlined in the hypotheses statements then this would omit the need to include statistical selection of variables into the model.

# Hypothesis testing (Stage 2)

Different mechanisms of AF should translate into different patterns of recurrence, and therefore into different responses to rhythm control therapy (Fabritz et al., 2016). The following seven *a priori* hypotheses were formulated based on published research.

1. The recurrence of AF differs in patients with and without a genetic or genomic predisposition to AF(Roselli et al., 2018) (defined as AF occurring first in those aged < 60 or with a family history of AF). In addition, we hypothesise that sodium channel blockers are more effective in preventing AF recurrence than other antiarrhythmic drugs in patients with a genetic or genomic predisposition to AF. This is based on experimental data on PITX2 levels and on the resting membrane potential(Syeda et al., 2016).
2. The recurrence of AF is more common in patients with concomitant heart failure than in those without heart failure (defined as elevated BNP, a surrogate marker for heart failure(Levin et al., 1998)). For patients with heart failure, we hypothesise that catheter ablation is more effective than antiarrhythmic drugs at preventing AF recurrence (Hsu et al., 2004, Khan et al., 2008, Marrouche et al., 2018).
3. The recurrence of AF is more common in obese patients (BMI ≥ 30). There is experimental and clinical evidence that increased fatty infiltration and activation of adipocytes in the atria cause AF(Haemers et al., 2015, Venteclef et al., 2013, Fabritz et al., 2016, Suffee et al., 2017), while weight reduction reduces recurrent AF in obese patients with AF(Pathak et al., 2015, Wong et al., 2011).
4. The recurrence of AF is more common in patients with severe hypertension (defined as left ventricular hypertrophy on echocardiogram or uncontrolled hypertension at baseline (blood pressure ≥ 160/90))(Schotten et al., 2011).
5. The recurrence of AF is more common in patients with chronic kidney disease (defined as elevated levels of fibroblast growth factor-23 as a surrogate marker for atrial fibrosis (Seiler et al., 2011, Geach, 2014, Chua et al., 2019)).
6. The prevalence of AF is associated with exercise intensity, which has been shown to modulate the relationship between physical activity and AF. Exercise load has been shown to correlate with AF incidence through a U-shaped curve(Guasch and Mont, 2017).
7. The prevalence of AF is associated with height. Preliminary data suggests that the autonomic tone could mediate this relationship. The association between stature, sex, heart rate and AF will be tested.

## Analytical approach

Hypothesis statements 1-5 will be assessed using separate Cox regression models with the outcome as time to recurrence of AF. Alongside the specific variables according to the statement, these models will include the prognostic index (see Table 1) for each patient obtained from the recurrence of AF model (optimism adjusted model) as an offset term to adjust for differences in case-mix. We will additionally include a term for each study to allow for differences in baseline risk. Hypothesis statements 6 and 7 will be assessed using separate logistic regression models with the outcome as AF status. Likewise, alongside the specific variables according to the statement, these models will include the prognostic index as an offset term and terms for each study to adjust for differences in baseline risk. These hypothesis tests will be performed on complete data.

The specific variables to be included in each of the models with regards to their hypothesis statement are as follows:

1. For statement 1, genetic or genomic predisposition to AF and treatment will be included in the model alongside the interaction between them.
2. For statement 2, heart failure and treatment will be included in the model alongside the interaction between them.
3. For statement 3, BMI dichotomised at 30 will be included in the model.
4. For statement 4, severe hypertension defined as either left ventricular hypertrophy or blood pressure ≥ 160/90 will be included in the model.
5. For statement 5, chronic kidney disease will be included in the model.
6. For statement 6, any form of participation in exercise will be included in the model.
7. For statement 7, height, gender and blood pressure will be included in the model.

# Reporting

For all of the models found in this study the full model including coefficients will be documented. The coefficients in the model will be displayed after they have been adjusted for optimism.

## Baseline characteristics

The study population baseline characteristics will be tabulated as per Tables A7-A11 in the Appendix. Categorical data will be summarised by frequencies and percentages. Continuous data will be summarised by the number of responses, mean and standard deviation if deemed to be normally distributed and number of responses, median and interquartile range if data appear skewed. Univariate analysis will also be conducted and included in this table. Each prognostic factor for each model will be summarised in a univariable way in the baseline characteristic table (Table A7-A11).

## Primary outcomes

A template of tables for reporting the results for both primary outcomes are documented in the Appendix (Tables A12-A15). These tables include the prognostic factors used in the models adjusted for each factor on each of the outcomes. The results from the apparent models and full optimism adjusted model for the two primary outcomes will be reported including the coeffiecients/OR/HR with corresponding 95% confidence intervals and p-values. For the optimism adjusted model with the outcome of time to recurrence of AF the baseline survival at 1 year will be also reported. The optimism adjusted model for prevalent AF will also report the constant for this model. All multivariable analysis will be adjusted for by each prognostic factor included in that model.

## Secondary outcomes

A template of tables for reporting the results for all secondary outcomes are documented in the Appendix (Tables A16-A21). These tables include the prognostic factors used in the models adjusted for each factor on each of the outcomes. These will contain HR and their corresponding 95% confidence intervals and p values for the Cox regression used. All multivariable analysis will be adjusted for by each prognostic factor included in that model.

## Levels of confidence intervals

Unless otherwise specified, estimates of differences between groups will be presented with 95%, two-sided confidence intervals.

# Limitations

Due to the retrospective nature of the study data and the combination of studies data, missing data will be inevitable. This is not ideal but using multiple imputation correctly will minimise this issue. Also the dichotomisation or categorisation of variables that could have remained continuous but haven’t due to the combining of study is sub-optimal from a statistical view. This will lead to the loss of information for these variables hence a less accurate representation of the variable on the outcome of interest.

# External validation of the prognostic models (WP4)

## Aims and objectives

The aim of WP4 is to externally validate the developed and optimism adjusted prognostic model for recurrence of AF in a separate dataset. This will determine how well the model works in a different population of patients to determine how transportable our model is.

The main objectives for WP4 are to:

1. Assess differences between the prognostic factor data used to develop the recurrence of AF model and the external validation data.
2. Assess the differences between the prognostic factor data used to develop the prevalent AF model and the external validation data.
3. External validate the recurrence of AF model using the AXAFA study data. This will be completed by fitting the model to the patient data in this separate dataset to establish the calibration and discrimination values with regards to performance of the model.
4. External validate the prevalent AF model using the UK Biobank data. This will be completed by fitting the model to the patient data in this separate dataset to establish the calibration and discrimination values with regards to performance of the model.
5. Compare the optimism adjusted recurrence of AF model to other clinically established prognostic models in use to understand how well our developed model works in relation to these models.

## Methods

### External validation

In the first instance the characteristics for the separate external validation data, this will be summarised and compared to the data used to develop the recurrence of AF model, this will form the baseline table. Most importantly the covariates in the external dataset will be checked to assess if they are in the same format and on the same scale as the development data to ensure that the external validation process is accurate when producing performance measures.

The external validation data will be assessed for missing data given the set of prognostic factors in the developed prognostic model. If there is 10% or more missing data then multiple imputation will be used in the same way as outlined in the development model methodology.

To complete the external validation for the developed model for recurrence of AF and prevalent AF we require the development model coefficients. This in turn will be used to fit to the data in the external validation data. The original optimism adjusted model will be assessed in this external dataset in the form of a c-statistic and corresponding 95% confidence value (discrimination) to establish how well our model works in this new separate dataset. Additionally a calibration plot displaying the predicted vs observed probability of having a recurrence of AF will be produced to establish how well the models work, likewise for the prevalent AF model.

The results of the calibration plot, c-statistic and corresponding 95% confidence interval will allow us to understand how well our optimism adjusted model for recurrence of AF working in a different population.

### Model comparisons

We will also compare the performance of the optimism adjusted model for recurrent AF with 4 existing scores: the CHADS_2_ score(Gage et al., 2001), the HATCH score(de Vos et al., 2010), the APPLE score(Kornej et al., 2015), and the ATLAS score(Mesquita et al., 2017). This will be completed by assessing the ROC curve in each case comparing to our developed model.

# Statistical software

STATA version 14 (or higher) will be used for all analyses.

# References

BURGESS, S., WHITE, I. R., RESCHE-RIGON, M. & WOOD, A. M. 2013. Combining multiple imputation and meta-analysis with individual participant data. *Stat Med,* 32**,** 4499-514.

CHUA, W., PURMAH, Y., CARDOSO, V. R., GKOUTOS, G. V., TULL, S. P., NECULAU, G., THOMAS, M. R., KOTECHA, D., LIP, G. Y. H., KIRCHHOF, P. & FABRITZ, L. 2019. Data-driven discovery and validation of circulating blood-based biomarkers associated with prevalent atrial fibrillation.

DE VOS, C. B., PISTERS, R., NIEUWLAAT, R., PRINS, M. H., TIELEMAN, R. G., COELEN, R. J., VAN DEN HEIJKANT, A. C., ALLESSIE, M. A. & CRIJNS, H. J. 2010. Progression from paroxysmal to persistent atrial fibrillation clinical correlates and prognosis. *J Am Coll Cardiol,* 55**,** 725-31.

FABRITZ, L., GUASCH, E., ANTONIADES, C., BARDINET, I., BENNINGER, G., BETTS, T. R., BRAND, E., BREITHARDT, G., BUCKLAR-SUCHANKOVA, G., CAMM, A. J., CARTLIDGE, D., CASADEI, B., CHUA, W. W., CRIJNS, H. J., DEEKS, J., HATEM, S., HIDDEN-LUCET, F., KAAB, S., MANIADAKIS, N., MARTIN, S., MONT, L., REINECKE, H., SINNER, M. F., SCHOTTEN, U., SOUTHWOOD, T., STOLL, M., VARDAS, P., WAKILI, R., WEST, A., ZIEGLER, A. & KIRCHHOF, P. 2016. Expert consensus document: Defining the major health modifiers causing atrial fibrillation: a roadmap to underpin personalized prevention and treatment. *Nat Rev Cardiol,* 13**,** 230-7.

GAGE, B. F., WATERMAN, A. D., SHANNON, W., BOECHLER, M., RICH, M. W. & RADFORD, M. J. 2001. Validation of clinical classification schemes for predicting stroke: Results from the national registry of atrial fibrillation. *JAMA,* 285**,** 2864-2870.

GEACH, T. 2014. Atrial fibrillation: FGF-23 associated with incident AF--a link with CKD? *Nat Rev Cardiol,* 11**,** 436.

GUASCH, E. & MONT, L. 2017. Diagnosis, pathophysiology, and management of exercise-induced arrhythmias. *Nat Rev Cardiol,* 14**,** 88-101.

HAEMERS, P., HAMDI, H., GUEDJ, K., SUFFEE, N., FARAHMAND, P., POPOVIC, N., CLAUS, P., LEPRINCE, P., NICOLETTI, A., JALIFE, J., WOLKE, C., LENDECKEL, U., JAIS, P., WILLEMS, R. & HATEM, S. N. 2015. Atrial fibrillation is associated with the fibrotic remodelling of adipose tissue in the subepicardium of human and sheep atria. *Eur Heart J*.

HEERINGA, J., VAN DER KUIP, D. A., HOFMAN, A., KORS, J. A., VAN HERPEN, G., STRICKER, B. H., STIJNEN, T., LIP, G. Y. & WITTEMAN, J. C. 2006. Prevalence, incidence and lifetime risk of atrial fibrillation: the Rotterdam study. *Eur Heart J,* 27**,** 949-53.

HSU, L. F., JAIS, P., SANDERS, P., GARRIGUE, S., HOCINI, M., SACHER, F., TAKAHASHI, Y., ROTTER, M., PASQUIE, J. L., SCAVEE, C., BORDACHAR, P., CLEMENTY, J. & HAISSAGUERRE, M. 2004. Catheter ablation for atrial fibrillation in congestive heart failure. *N Engl J Med,* 351**,** 2373-83.

KHAN, M. N., JAIS, P., CUMMINGS, J., DI BIASE, L., SANDERS, P., MARTIN, D. O., KAUTZNER, J., HAO, S., THEMISTOCLAKIS, S., FANELLI, R., POTENZA, D., MASSARO, R., WAZNI, O., SCHWEIKERT, R., SALIBA, W., WANG, P., AL-AHMAD, A., BEHEIRY, S., SANTARELLI, P., STARLING, R. C., DELLO RUSSO, A., PELARGONIO, G., BRACHMANN, J., SCHIBGILLA, V., BONSO, A., CASELLA, M., RAVIELE, A., HAISSAGUERRE, M. & NATALE, A. 2008. Pulmonary-vein isolation for atrial fibrillation in patients with heart failure. *N Engl J Med,* 359**,** 1778-85.

KORNEJ, J., HINDRICKS, G., SHOEMAKER, M. B., HUSSER, D., ARYA, A., SOMMER, P., ROLF, S., SAAVEDRA, P., KANAGASUNDRAM, A., PATRICK WHALEN, S., MONTGOMERY, J., ELLIS, C. R., DARBAR, D. & BOLLMANN, A. 2015. The APPLE score: a novel and simple score for the prediction of rhythm outcomes after catheter ablation of atrial fibrillation. *Clin Res Cardiol,* 104**,** 871-6.

LEVIN, E. R., GARDNER, D. G. & SAMSON, W. K. 1998. Natriuretic Peptides. *New England Journal of Medicine,* 339**,** 321-328.

MARROUCHE, N. F., BRACHMANN, J., ANDRESEN, D., SIEBELS, J., BOERSMA, L., JORDAENS, L., MERKELY, B., POKUSHALOV, E., SANDERS, P., PROFF, J., SCHUNKERT, H., CHRIST, H., VOGT, J., BANSCH, D. & INVESTIGATORS, C.-A. 2018. Catheter Ablation for Atrial Fibrillation with Heart Failure. *N Engl J Med,* 378**,** 417-427.

MESQUITA, J., FERREIRA, A. M., CAVACO, D., MOSCOSO COSTA, F., CARMO, P., MARQUES, H., MORGADO, F., MENDES, M. & ADRAGAO, P. 2017. Development and validation of a risk score for predicting atrial fibrillation recurrence after a first catheter ablation procedure - ATLAS score. *Europace*.

MORRIS, T. P., WHITE, I. R., CARPENTER, J. R., STANWORTH, S. J. & ROYSTON, P. 2015. Combining fractional polynomial model building with multiple imputation. *Stat Med,* 34**,** 3298-317.

MORRIS, T. P., WHITE, I. R., ROYSTON, P., SEAMAN, S. R. & WOOD, A. M. 2014. Multiple imputation for an incomplete covariate that is a ratio. *Stat Med,* 33**,** 88-104.

PATHAK, R. K., MIDDELDORP, M. E., MEREDITH, M., MEHTA, A. B., MAHAJAN, R., WONG, C. X., TWOMEY, D., ELLIOTT, A. D., KALMAN, J. M., ABHAYARATNA, W. P., LAU, D. H. & SANDERS, P. 2015. Long-Term Effect of Goal-Directed Weight Management in an Atrial Fibrillation Cohort: A Long-Term Follow-Up Study (LEGACY). *J Am Coll Cardiol,* 65**,** 2159-69.

PEDUZZI, P., CONCATO, J., KEMPER, E., HOLFORD, T. R. & FEINSTEIN, A. R. 1996. A simulation study of the number of events per variable in logistic regression analysis. *J Clin Epidemiol,* 49.

ROSELLI, C., CHAFFIN, M. D., WENG, L. C., AESCHBACHER, S., AHLBERG, G., ALBERT, C. M., ALMGREN, P., ALONSO, A., ANDERSON, C. D., ARAGAM, K. G., ARKING, D. E., BARNARD, J., BARTZ, T. M., BENJAMIN, E. J., BIHLMEYER, N. A., BIS, J. C., BLOOM, H. L., BOERWINKLE, E., BOTTINGER, E. B., BRODY, J. A., CALKINS, H., CAMPBELL, A., CAPPOLA, T. P., CARLQUIST, J., CHASMAN, D. I., CHEN, L. Y., CHEN, Y. I., CHOI, E. K., CHOI, S. H., CHRISTOPHERSEN, I. E., CHUNG, M. K., COLE, J. W., CONEN, D., COOK, J., CRIJNS, H. J., CUTLER, M. J., DAMRAUER, S. M., DANIELS, B. R., DARBAR, D., DELGADO, G., DENNY, J. C., DICHGANS, M., DORR, M., DUDINK, E. A., DUDLEY, S. C., ESA, N., ESKO, T., ESKOLA, M., FATKIN, D., FELIX, S. B., FORD, I., FRANCO, O. H., GEELHOED, B., GREWAL, R. P., GUDNASON, V., GUO, X., GUPTA, N., GUSTAFSSON, S., GUTMANN, R., HAMSTEN, A., HARRIS, T. B., HAYWARD, C., HECKBERT, S. R., HERNESNIEMI, J., HOCKING, L. J., HOFMAN, A., HORIMOTO, A., HUANG, J., HUANG, P. L., HUFFMAN, J., INGELSSON, E., IPEK, E. G., ITO, K., JIMENEZ-CONDE, J., JOHNSON, R., JUKEMA, J. W., KAAB, S., KAHONEN, M., KAMATANI, Y., KANE, J. P., KASTRATI, A., KATHIRESAN, S., KATSCHNIG-WINTER, P., KAVOUSI, M., KESSLER, T., KIETSELAER, B. L., KIRCHHOF, P., KLEBER, M. E., KNIGHT, S., KRIEGER, J. E., KUBO, M., LAUNER, L. J., LAURIKKA, J., LEHTIMAKI, T., LEINEWEBER, K., LEMAITRE, R. N., LI, M., LIM, H. E., LIN, H. J., LIN, H., et al. 2018. Multi-ethnic genome-wide association study for atrial fibrillation. *Nat Genet*.

RUBIN, D. B. 1987. *Multiple Imputation for Nonresponse in Surveys,* New York, Sons.

RUBIN, D. B. 2008. Frontmatter. *Multiple Imputation for Nonresponse in Surveys.* John Wiley & Sons, Inc.

SAUERBREI, W. 1999. The use of resampling methods to simplify regression models in medical statistics. *Applied Statistics,* 48.

SCHOTTEN, U., VERHEULE, S., KIRCHHOF, P. & GOETTE, A. 2011. Pathophysiological mechanisms of atrial fibrillation: a translational appraisal. *Physiol Rev,* 91**,** 265-325.

SEAMAN, S. R., BARTLETT, J. W. & WHITE, I. R. 2012. Multiple imputation of missing covariates with non-linear effects and interactions: an evaluation of statistical methods. *BMC Med Res Methodol,* 12**,** 46.

SEILER, S., CREMERS, B., REBLING, N. M., HORNOF, F., JEKEN, J., KERSTING, S., STEIMLE, C., EGE, P., FEHRENZ, M., ROGACEV, K. S., SCHELLER, B., BOHM, M., FLISER, D. & HEINE, G. H. 2011. The phosphatonin fibroblast growth factor 23 links calcium-phosphate metabolism with left-ventricular dysfunction and atrial fibrillation. *Eur Heart J,* 32**,** 2688-96.

SUFFEE, N., MOORE-MORRIS, T., FARAHMAND, P., RUCKER-MARTIN, C., DILANIAN, G., FRADET, M., SAWAKI, D., DERUMEAUX, G., LEPRINCE, P., CLEMENT, K., DUGAIL, I., PUCEAT, M. & HATEM, S. N. 2017. Atrial natriuretic peptide regulates adipose tissue accumulation in adult atria. *Proc Natl Acad Sci U S A,* 114**,** E771-E780.

SYEDA, F., HOLMES, A. P., YU, T. Y., TULL, S., KUHLMANN, S. M., PAVLOVIC, D., BETNEY, D., RILEY, G., KUCERA, J. P., JOUSSET, F., DE GROOT, J. R., ROHR, S., BROWN, N., FABRITZ, L. & KIRCHHOF, P. 2016. PITX2 modulates atrial membrane potential and reduced PITX2 potentiates the antiarrhythmic effects of sodium-channel blockers. *JACC,* 68**,** 59-72; doi: 10.1016/j.jacc.2016.07.766.

VENTECLEF, N., GUGLIELMI, V., BALSE, E., GABORIT, B., COTILLARD, A., ATASSI, F., AMOUR, J., LEPRINCE, P., DUTOUR, A., CLEMENT, K. & HATEM, S. N. 2013. Human epicardial adipose tissue induces fibrosis of the atrial myocardium through the secretion of adipo-fibrokines. *Eur Heart J*.

WHITE, I. R., ROYSTON, P. & WOOD, A. M. 2011. Multiple imputation using chained equations: Issues and guidance for practice. *Statistics in Medicine,* 30**,** 377-399.

WONG, C. X., ABED, H. S., MOLAEE, P., NELSON, A. J., BROOKS, A. G., SHARMA, G., LEONG, D. P., LAU, D. H., MIDDELDORP, M. E., ROBERTS-THOMSON, K. C., WITTERT, G. A., ABHAYARATNA, W. P., WORTHLEY, S. G. & SANDERS, P. 2011. Pericardial fat is associated with atrial fibrillation severity and ablation outcome. *J Am Coll Cardiol,* 57**,** 1745-51.

WOOD, A. M., WHITE, I. R. & ROYSTON, P. 2008. How should variable selection be performed with multiply imputed data? *Stat Med,* 27**,** 3227-46.

# Appendix

## Study characteristics table

|  | Studies in the CATCH ME database | | | | | | | | | | | | | |
| --- | --- | --- | --- | --- | --- | --- | --- | --- | --- | --- | --- | --- | --- | --- |
| Study Characteristics | **AFCT** | **AFLMU** | **BBC-AF** | **Flec-SL** | **FUTURE** | **GIRAFA** | **MULTI-AF** | **Maastricht tissue bank** | **PVIBCN** | **RACE4** | | **READ-POAF** | | **KORA** |
| Study objectives |  |  |  |  |  |  |  |  |  | |  | |  |  |
| Study population |  |  |  |  |  |  |  |  |  | |  | |  |  |
| Inclusion/  exclusion criteria |  |  |  |  |  |  |  |  |  | |  | |  |  |
| Randomisation |  |  |  |  |  |  |  |  |  | |  | |  |  |
| Location of study |  |  |  |  |  |  |  |  |  | |  | |  |  |
| Data collection |  |  |  |  |  |  |  |  |  | |  | |  |  |
| Year of study |  |  |  |  |  |  |  |  |  | |  | |  |  |
| Duration of study |  |  |  |  |  |  |  |  |  | |  | |  |  |
| Follow-up time |  |  |  |  |  |  |  |  |  | |  | |  |  |
| Biomarkers |  |  |  |  |  |  |  |  |  | |  | |  |  |
| Type of study |  |  |  |  |  |  |  |  |  | |  | |  |  |
| Number of events |  |  |  |  |  |  |  |  |  | |  | |  |  |
| Number of participants |  |  |  |  |  |  |  |  |  | |  | |  |  |
| Time to event data |  |  |  |  |  |  |  |  |  | |  | |  |  |

Table A1: Displaying the characteristics of each study that has contributed to the data for the CATCH ME database.

## Sample size tables for each outcome

### Prevalent AF

| Study ID | No. of participants | No. of participants at follow up | No. of events |
| --- | --- | --- | --- |
| Study # |  |  |  |
| Study # |  |  |  |
| Study # |  |  |  |
| Study # |  |  |  |
| … |  |  |  |

**Table A2:** Sample size table for the outcome of prevelant AF.

### Recurrence of AF

| Study ID | No. of participants | No. of participants at follow up | No. of events |
| --- | --- | --- | --- |
| Study # |  |  |  |
| Study # |  |  |  |
| Study # |  |  |  |
| Study # |  |  |  |
| … |  |  |  |

**Table A3:** Sample size table for the outcome of recurrence of AF.

### Stroke

| Study ID | No. of participants | No. of participants at follow up | No. of events |
| --- | --- | --- | --- |
| Study # |  |  |  |
| Study # |  |  |  |
| Study # |  |  |  |
| Study # |  |  |  |
| … |  |  |  |

**Table A4:** Sample size table for the outcome of stroke.

### Cardiovascular death

| Study ID | No. of participants | No. of participants at follow up | No. of events |
| --- | --- | --- | --- |
| Study # |  |  |  |
| Study # |  |  |  |
| Study # |  |  |  |
| Study # |  |  |  |
| … |  |  |  |

**Table A5:** Sample size table for the outcome of cardiovascular death.

### Worsening of heart failure

| Study ID | No. of participants | No. of participants at follow up | No. of events |
| --- | --- | --- | --- |
| Study # |  |  |  |
| Study # |  |  |  |
| Study # |  |  |  |
| Study # |  |  |  |
| … |  |  |  |

**Table A6:** Sample size table for the outcome of worsening of heart failure.

## Baseline characteristics table

### Primary outcomes

#### Prevalent AF

| Candidate predictor | No first diagnosis of AF (N=)  (No Event) | First diagnosis of AF (N=)  (Event) | Hazard Ratio (95% Confidence interval) | p-value | Missing data (%) |
| --- | --- | --- | --- | --- | --- |
| Age(years) |  |  |  |  |  |
| Gender |  |  |  |  |  |
| Study |  |  |  |  |  |
| Race |  |  |  |  |  |
| BMI |  |  |  |  |  |
| biomarker A |  |  |  |  |  |
| biomarker B |  |  |  |  |  |
| …… |  |  |  |  |  |

For continuous predictors the Mean (SD) or Median (IQR) dependent on data distribution

**Table A7:** Displaying the baseline characteristics found using the Delphi process plus the biomarkers found in WP1 and WP2 with the data split by outcome (event vs non-event). Univariate analysis displaying the hazard ratio and its corresponding 95% confidence interval and p-value.

#### Recurrence of AF

| Candidate predictor | No Recurrence of AF (N=)  (No Event) | Recurrence of AF (N=)  (Event) | Hazard Ratio (95% Confidence interval) | p-value | Missing data (%) |
| --- | --- | --- | --- | --- | --- |
| Age(years) |  |  |  |  |  |
| Gender |  |  |  |  |  |
| Study |  |  |  |  |  |
| Race |  |  |  |  |  |
| BMI |  |  |  |  |  |
| biomarker A |  |  |  |  |  |
| biomarker B |  |  |  |  |  |
| …… |  |  |  |  |  |

For continuous predictors the Mean (SD) or Median (IQR) dependent on data distribution

**Table A8**: Displaying the baseline characteristics found using the Delphi process plus the biomarkers found in WP1 and WP2 with the data split by outcome (event vs non-event). Univariate analysis displaying the hazard ratio and its corresponding 95% confidence interval and p-value.

### Secondary outcomes

#### Stroke

| Candidate predictor | No Stroke (N=)  (No Event) | Stroke (N=)  (Event) | Hazard Ratio (95% Confidence interval) | p-value | Missing data (%) |
| --- | --- | --- | --- | --- | --- |
| Age(years) |  |  |  |  |  |
| Gender |  |  |  |  |  |
| Study |  |  |  |  |  |
| Race |  |  |  |  |  |
| BMI |  |  |  |  |  |
| biomarker A |  |  |  |  |  |
| biomarker B |  |  |  |  |  |
| …… |  |  |  |  |  |

For continuous predictors the Mean (SD) or Median (IQR) dependent on data distribution

**Table A9**: Displaying the baseline characteristics found using the Delphi process plus the biomarkers found in WP1 and WP2 with the data split by outcome (event vs non-event). Univariate analysis displaying the hazard ratio and its corresponding 95% confidence interval and p-value.

#### Cardiovascular death

| Candidate predictor | No Cardiovascular death (N=)  (No Event) | Cardiovascular death (N=)  (Event) | Hazard Ratio (95% Confidence interval) | p-value | Missing data (%) |
| --- | --- | --- | --- | --- | --- |
| Age(years) |  |  |  |  |  |
| Gender |  |  |  |  |  |
| Study |  |  |  |  |  |
| Race |  |  |  |  |  |
| BMI |  |  |  |  |  |
| biomarker A |  |  |  |  |  |
| biomarker B |  |  |  |  |  |
| …… |  |  |  |  |  |

For continuous predictors the Mean (SD) or Median (IQR) dependent on data distribution

**Table A10**: Displaying the baseline characteristics found using the Delphi process plus the biomarkers found in WP1 and WP2 with the data split by outcome (event vs non-event). Univariate analysis displaying the hazard ratio and its corresponding 95% confidence interval and p-value.

#### Worsening of heart failure

| Candidate predictor | No Worsening of heart failure(N=)  (No Event) | Worsening of heart failure (N=)  (Event) | Hazard Ratio (95% Confidence interval) | p-value | Missing data (%) |
| --- | --- | --- | --- | --- | --- |
| Age(years) |  |  |  |  |  |
| Gender |  |  |  |  |  |
| Study |  |  |  |  |  |
| Race |  |  |  |  |  |
| BMI |  |  |  |  |  |
| biomarker A |  |  |  |  |  |
| biomarker B |  |  |  |  |  |
| …… |  |  |  |  |  |

For continuous predictors the Mean (SD) or Median (IQR) dependent on data distribution

**Table A11**: Displaying the baseline characteristics found using the Delphi process plus the biomarkers found in WP1 and WP2 with the data split by outcome (event vs non-event). Univariate analysis displaying the hazard ratio and its corresponding 95% confidence interval and p-value.

## Primary outcome results

All models will have 2 tables per outcome: (a) with imputed values and (b) complete case data (i.e. with no imputed data)

### Prevalent AF

| Model 1 - Adjusted analysis for prevelant AF | | | |
| --- | --- | --- | --- |
| Characteristic | Hazard Ratio | 95% Confidence interval | P value |
| Age (years) |  |  |  |
| Gender |  |  |  |
| Study |  |  |  |
| … |  |  |  |

**Table A12**: Displaying the results of Model 1 for each characteristic found using the Delphi process and selected by the stepwise procedure for the adjusted analysis on the prevalent AF. The results are produced in the form of hazards ratios with corresponding 95% confidence intervals and P values.

| Model 2 - Adjusted analysis for prevelant AF | | | |
| --- | --- | --- | --- |
| Characteristic | Hazard Ratio | 95% Confidence interval | P value |
| Age (years) |  |  |  |
| Gender |  |  |  |
| Study  Biomarker A  Biomarker B |  |  |  |
| … |  |  |  |

**Table A13**: Displaying the results of Model 2 for each characteristic found using the Delphi process and selected by the stepwise procedure with the addition of the biomarkers for the adjusted analysis on the prevalent AF. The results are produced in the form of hazards ratios with corresponding 95% confidence intervals and P values.

### Recurrence of AF

| Model 1 - Adjusted analysis for Recurrent AF | | | |
| --- | --- | --- | --- |
| Characteristic | Hazard Ratio | 95% Confidence interval | P value |
| Age (years) |  |  |  |
| Gender |  |  |  |
| Study |  |  |  |
| … |  |  |  |

**Table A14**: Displaying the results of Model 1 for each characteristic found using the Delphi process and selected by the stepwise procedure for the adjusted analysis on recurrence of AF. The results are produced in the form of hazards ratios with corresponding 95% confidence intervals and P values.

| Model 2 - Adjusted analysis for Recurrent AF | | | |
| --- | --- | --- | --- |
| Characteristic | Hazard Ratio | 95% Confidence interval | P value |
| Age (years) |  |  |  |
| Gender |  |  |  |
| Study  Biomarker A  Biomarker B |  |  |  |
| … |  |  |  |

**Table A15**: Displaying the results of Model 2 for each characteristic found using the Delphi process and selected by the stepwise procedure with the addition of the biomarkers for the adjusted analysis on recurrence of AF. The results are produced in the form of hazards ratios with corresponding 95% confidence intervals and P values.

## Secondary outcome results

All models will have 2 tables per outcome: (a) with imputed values and (b) complete case data (i.e. with no imputed data)

### Stroke

| Model 1 - Adjusted analysis for Stroke | | | |
| --- | --- | --- | --- |
| Characteristic | Hazard Ratio | 95% Confidence interval | P value |
| Age (years) |  |  |  |
| Gender |  |  |  |
| Study |  |  |  |
| … |  |  |  |

**Table A16**: Displaying the results of Model 1 for each characteristic found using the Delphi process and selected by the stepwise procedure for the adjusted analysis on stroke. The results are produced in the form of hazards ratios with corresponding 95% confidence intervals and P values.

| Model 2 - Adjusted analysis for Stroke | | | |
| --- | --- | --- | --- |
| Characteristic | Hazard Ratio | 95% Confidence interval | P value |
| Age (years) |  |  |  |
| Gender |  |  |  |
| Study  Biomarker A  Biomarker B |  |  |  |
| … |  |  |  |

**Table A17:** Displaying the results of Model 2 for each characteristic found using the Delphi process and selected by the stepwise procedure with the addition of the biomarkers for the adjusted analysis on stroke. The results are produced in the form of hazards ratios with corresponding 95% confidence intervals and P values.

### Cardiovascular death

| Model 1 - Adjusted analysis for Cardiovascular death | | | |
| --- | --- | --- | --- |
| Characteristic | Hazard Ratio | 95% Confidence interval | P value |
| Age (years) |  |  |  |
| Gender |  |  |  |
| Study |  |  |  |
| … |  |  |  |

**Table A18**: Displaying the results of Model 1 for each characteristic found using the Delphi process and selected by the stepwise procedure for the adjusted analysis on cardiovascular death. The results are produced in the form of hazards ratios with corresponding 95% confidence intervals and P values.

| Model 2 - Adjusted analysis for Cardiovascular death | | | |
| --- | --- | --- | --- |
| Characteristic | Hazard Ratio | 95% Confidence interval | P value |
| Age (years) |  |  |  |
| Gender |  |  |  |
| Study  Biomarker A  Biomarker B |  |  |  |
| … |  |  |  |

**Table A19**: Displaying the results of Model 2 for each characteristic found using the Delphi process and selected by the stepwise procedure with the addition of the biomarkers for the adjusted analysis on cardiovascular death. The results are produced in the form of hazards ratios with corresponding 95% confidence intervals and P values.

### Worsening of heart failure

| Model 1 - Adjusted analysis for Worsening of heart failure | | | |
| --- | --- | --- | --- |
| Characteristic | Hazard Ratio | 95% Confidence interval | P value |
| Age (years) |  |  |  |
| Gender |  |  |  |
| Study |  |  |  |
| … |  |  |  |

**Table A20**: Displaying the results of Model 1 for each characteristic found using the Delphi process and selected by the stepwise procedure for the adjusted analysis on the worsening of heart failure. The results are produced in the form of hazards ratios with corresponding 95% confidence intervals and P values.

| Model 2 - Adjusted analysis for Worsening of heart failure | | | |
| --- | --- | --- | --- |
| Characteristic | Hazard Ratio | 95% Confidence interval | P value |
| Age (years) |  |  |  |
| Gender |  |  |  |
| Study  Biomarker A  Biomarker B |  |  |  |
| … |  |  |  |

**Table A21**: Displaying the results of Model 2 for each characteristic found using the Delphi process and selected by the stepwise procedure with the addition of the biomarkers for the adjusted analysis on the worsening of heart failure. The results are produced in the form of hazards ratios with corresponding 95% confidence intervals and P values.

## Equations for all models produced

General format for survival analysis equation using Cox regression:

S(t) = S_0_ (t) ^exp ((β_1_*X_1_ +⋯+ β_n_*X_n_))

S_0_ (t) is the baseline hazard

General format for logistic regression:

Log odds = β_0_+(β_1_*X_1_ )+⋯+ (β_n_*X_n_)

Where the log odds are log(p/1-p); p=probability of event

Each model that has been developed will be documented using the general format above, including the coefficients for each of the covariates in the model.

## Model performance tables

All models will have 2 tables per outcome: (a) with imputed values and (b) complete case data (i.e. with no imputed data)

### Primary outcomes

#### Prevalent AF

|  | Prevalent AF probability cut-off value | | | |
| --- | --- | --- | --- | --- |
| Model 1 |  | | | |
|  | A%* | B%* | C%* | D%* |
| Sensitivity (%) |  |  |  |  |
| Specificity (%) |  |  |  |  |
| Positive Predictive Value (%) |  |  |  |  |
| Negative predictive value (%) |  |  |  |  |
| Number of participants |  |  |  |  |
| Number who had a first diagnosis of AF |  |  |  |  |
| Number deemed at risk |  |  |  |  |
| Number “identified” |  |  |  |  |
| Number deemed not at risk |  |  |  |  |
| Number “missed” |  |  |  |  |

* Clinically chosen cut points

**Table A22**: Displaying the model performance results of Model 1 for each of the clinically chosen cut-off points for the prevalent AF.

|  | Prevalent AF probability cut-off value | | | |
| --- | --- | --- | --- | --- |
| Model 2 |  | | | |
|  | A%* | B%* | C%* | D%* |
| Sensitivity (%) |  |  |  |  |
| Specificity (%) |  |  |  |  |
| Positive Predictive Value (%) |  |  |  |  |
| Negative predictive value (%) |  |  |  |  |
| Number of participants |  |  |  |  |
| Number who had a first diagnosis of AF |  |  |  |  |
| Number deemed at risk |  |  |  |  |
| Number “identified” |  |  |  |  |
| Number deemed not at risk |  |  |  |  |
| Number “missed” |  |  |  |  |

* Clinically chosen cut points

**Table A23**: Displaying the model performance results of Model 2 for each of the clinically chosen cut-off points for the prevalent AF.

#### Recurrence of AF

|  | Recurrence of AF probability cut-off value | | | |
| --- | --- | --- | --- | --- |
| Model 1 |  | | | |
|  | A%* | B%* | C%* | D%* |
| Sensitivity (%) |  |  |  |  |
| Specificity (%) |  |  |  |  |
| Positive Predictive Value (%) |  |  |  |  |
| Negative predictive value (%) |  |  |  |  |
| Number of participants |  |  |  |  |
| Number who had a recurrence of AF |  |  |  |  |
| Number deemed at risk |  |  |  |  |
| Number “identified” |  |  |  |  |
| Number deemed not at risk |  |  |  |  |
| Number “missed” |  |  |  |  |

* Clinically chosen cut points

**Table A24**: Displaying the model performance results of Model 1 for each of the clinically chosen cut-off points for the recurrence of AF.

|  | Recurrence of AF probability cut-off value | | | |
| --- | --- | --- | --- | --- |
| Model 2 |  | | | |
|  | A%* | B%* | C%* | D%* |
| Sensitivity (%) |  |  |  |  |
| Specificity (%) |  |  |  |  |
| Positive Predictive Value (%) |  |  |  |  |
| Negative predictive value (%) |  |  |  |  |
| Number of participants |  |  |  |  |
| Number who had a recurrence of AF |  |  |  |  |
| Number deemed at risk |  |  |  |  |
| Number “identified” |  |  |  |  |
| Number deemed not at risk |  |  |  |  |
| Number “missed” |  |  |  |  |

* Clinically chosen cut points

**Table A25**: Displaying the model performance results of Model 2 for each of the clinically chosen cut-off points for the recurrence of AF.

### Secondary outcomes

#### Stroke

|  | Stroke probability cut-off value | | | |
| --- | --- | --- | --- | --- |
| Model 1 |  | | | |
|  | A%* | B%* | C%* | D%* |
| Sensitivity (%) |  |  |  |  |
| Specificity (%) |  |  |  |  |
| Positive Predictive Value (%) |  |  |  |  |
| Negative predictive value (%) |  |  |  |  |
| Number of participants |  |  |  |  |
| Number who had a Stroke |  |  |  |  |
| Number deemed at risk |  |  |  |  |
| Number “identified” |  |  |  |  |
| Number deemed not at risk |  |  |  |  |
| Number “missed” |  |  |  |  |

* Clinically chosen cut points

**Table A26**: Displaying the model performance results of Model 1 for each of the clinically chosen cut-off points for stroke.

|  | Stroke probability cut-off value | | | |
| --- | --- | --- | --- | --- |
| Model 2 |  | | | |
|  | A%* | B%* | C%* | D%* |
| Sensitivity (%) |  |  |  |  |
| Specificity (%) |  |  |  |  |
| Positive Predictive Value (%) |  |  |  |  |
| Negative predictive value (%) |  |  |  |  |
| Number of participants |  |  |  |  |
| Number who had a Stroke |  |  |  |  |
| Number deemed at risk |  |  |  |  |
| Number “identified” |  |  |  |  |
| Number deemed not at risk |  |  |  |  |
| Number “missed” |  |  |  |  |

* Clinically chosen cut points

**Table A27**: Displaying the model performance results of Model 2 for each of the clinically chosen cut-off points for stroke.

#### Cardiovascular death

|  | Cardiovascular death probability cut-off value | | | |
| --- | --- | --- | --- | --- |
| Model 1 |  | | | |
|  | A%* | B%* | C%* | D%* |
| Sensitivity (%) |  |  |  |  |
| Specificity (%) |  |  |  |  |
| Positive Predictive Value (%) |  |  |  |  |
| Negative predictive value (%) |  |  |  |  |
| Number of participants |  |  |  |  |
| Number who had a cardiovascular death |  |  |  |  |
| Number deemed at risk |  |  |  |  |
| Number “identified” |  |  |  |  |
| Number deemed not at risk |  |  |  |  |
| Number “missed” |  |  |  |  |

* Clinically chosen cut points

**Table A28**: Displaying the model performance results of Model 1 for each of the clinically chosen cut-off points for cardiovascular death.

|  | Cardiovascular death probability cut-off value | | | |
| --- | --- | --- | --- | --- |
| Model 2 |  | | | |
|  | A%* | B%* | C%* | D%* |
| Sensitivity (%) |  |  |  |  |
| Specificity (%) |  |  |  |  |
| Positive Predictive Value (%) |  |  |  |  |
| Negative predictive value (%) |  |  |  |  |
| Number of participants |  |  |  |  |
| Number who had a cardiovascular death |  |  |  |  |
| Number deemed at risk |  |  |  |  |
| Number “identified” |  |  |  |  |
| Number deemed not at risk |  |  |  |  |
| Number “missed” |  |  |  |  |

* Clinically chosen cut points

**Table A29**: Displaying the model performance results of Model 2 for each of the clinically chosen cut-off points for cardiovascular death.

#### Worsening of heart failure

|  | Worsening of heart failure probability cut-off value | | | |
| --- | --- | --- | --- | --- |
| Model 1 |  | | | |
|  | A%* | B%* | C%* | D%* |
| Sensitivity (%) |  |  |  |  |
| Specificity (%) |  |  |  |  |
| Positive Predictive Value (%) |  |  |  |  |
| Negative predictive value (%) |  |  |  |  |
| Number of participants |  |  |  |  |
| Number who had a worsening of heart failure |  |  |  |  |
| Number deemed at risk |  |  |  |  |
| Number “identified” |  |  |  |  |
| Number deemed not at risk |  |  |  |  |
| Number “missed” |  |  |  |  |

* Clinically chosen cut points

**Table A30**: Displaying the model performance results of Model 1 for each of the clinically chosen cut-off points for worsening of heart failure.

|  | Worsening of heart failure probability cut-off value | | | |
| --- | --- | --- | --- | --- |
| Model 2 |  | | | |
|  | A%* | B%* | C%* | D%* |
| Sensitivity (%) |  |  |  |  |
| Specificity (%) |  |  |  |  |
| Positive Predictive Value (%) |  |  |  |  |
| Negative predictive value (%) |  |  |  |  |
| Number of participants |  |  |  |  |
| Number who had a worsening of heart failure |  |  |  |  |
| Number deemed at risk |  |  |  |  |
| Number “identified” |  |  |  |  |
| Number deemed not at risk |  |  |  |  |
| Number “missed” |  |  |  |  |

* Clinically chosen cut points

**Table A31:** Displaying the model performance results of Model 2 for each of the clinically chosen cut-off points for worsening of heart failure.

| Outcome | Prevalence of outcome |
| --- | --- |
| Primary outcome |  |
| Recurrence of AF |  |
| Secondary outcome |  |
| Stroke |  |
| Cardiovascular death |  |
| Worsening of heart failure |  |

**Table A32:** The prevalence of each of the primary and secondary outcomes displayed as percentages.

|  | | | Prediction | | |
| --- | --- | --- | --- | --- | --- |
| Actual |  | **Positive +** | | **Negative -** | **Total** |
|  | **Positive +** | Correctly identified  (True Positive, TP) | | Falsely unidentified  (False Negative, FN) | ∑ Condition is Positive (TP+FN) |
|  | **Negative -** | Falsely identified  (False positive, FP) | | Correctly identified  (True Negative, TN) | ∑ Condition is Negative (FP+TN) |
|  | **Total** | ∑ Test is Positive  (TP+FP) | | ∑ Test is Negative  (FN+TN) | ∑ All people  (TP+FN+TN+FP) |

**Table A33**: An example of a 2x2 table format to be completed for each model and outcome.

## Calibration

A Calibration plot will be constructed for each of Models 1 and 2. For Cox regression the Loess method will be implemented on the calibration plot.

## Discriminants

### Receiver operating characteristic curve

Sensitivity

1-Specificity

0.10

0.20

0.30

0.40

0.50

0.60

0.70

0.80

0.90

1.00

0.10

0.20

0.30

0.40

0.50

0.60

0.70

0.80

0.90

1.00

1% cut-off point

2% cut-off point

5% cut-off point

10% cut-off point

Figure A1: An illustrative ROC curve for the cut-off points: 1%, 2%, 5% and 10%. (Webb, S.S. et al. Int Urogynecol J (2016). doi:10.1007/s00192-016-3125-2). This is an example of the ROC that will be produced for each model on each outcome.

### Concordance-Statistic (C-statistic) table

Two tables will be created to report the C-statistic :(a) with imputed values and (b) complete case data (i.e. with no imputed data)

| Primary outcomes | Models | C-statistics  (95% Confidence Interval) |
| --- | --- | --- |
| Prevalent AF |  |  |
|  | Model 1 |  |
|  | Model 2 |  |
| Recurrence of AF |  |  |
|  | Model 1 |  |
|  | Model 2 |  |

| Secondary outcomes | Models | C-statistics  (95% Confidence Interval) |
| --- | --- | --- |
| Stroke |  |  |
|  | Model 1 |  |
|  | Model 2 |  |
| Cardiovascular death |  |  |
|  | Model 1 |  |
|  | Model 2 |  |
| Worsening of heart failure |  |  |
|  | Model 1 |  |
|  | Model 2 |  |

**Table A34**: Displaying the c-statistic values for each of the models under each of the outcomes with its corresponding 95% confidence interval.

## Missing data

|  | Missing data (%) for all candidate predictors identified by the Delphi survey plus the biomarkers | | | | | | | | | | |  | |  |
| --- | --- | --- | --- | --- | --- | --- | --- | --- | --- | --- | --- | --- | --- | --- |
| Candidate predictors | **Study name** | | | | | | | | | | |  | |  |
|  | AFCT | AFLMU | BBC-AF | Flec-SL | FUTURE | GIRAFA | MULTI-AF | | Maastricht tissue bank | PVIBCN | RACE4 | | READ-POAF | KORA |
| Age |  |  |  |  |  |  |  |  | |  |  |  | |  |
| Gender |  |  |  |  |  |  |  |  | |  |  |  | |  |
| Race |  |  |  |  |  |  |  |  | |  |  |  | |  |
| BMI |  |  |  |  |  |  |  |  | |  |  |  | |  |
| biomarker A |  |  |  |  |  |  |  |  | |  |  |  | |  |
| biomarker B |  |  |  |  |  |  |  |  | |  |  |  | |  |
| … |  |  |  |  |  |  |  |  | |  |  |  | |  |

Table A35: Displaying the percentage of missing data for each of the predictors found from the Delphi survey for each of the studies that are included in the CATCH ME database plus the addition of the biomarkers identified by WP1 and WP2.

## Kaplan-Meier estimators

Kaplan-Meier curves will be produced where Cox regression has been used. These curves will consist of plots for each primary and secondary outcome displaying curves for Model 1 and Model 2 in a single plot.
